# Perinatal insult dimensions and developmental trajectories of psychotic-like experiences

**DOI:** 10.1038/s41537-025-00662-6

**Published:** 2025-08-25

**Authors:** Eric R. Larson, Nicole R. Karcher, Alexandra B. Moussa-Tooks

**Affiliations:** 1 Department of Psychological & Brain Sciences, Indiana University, Bloomington, IN USA; 2 Program in Neuroscience, Indiana University, Bloomington, IN USA; 3 Department of Psychiatry, Washington University School of Medicine, St. Louis, MO USA; 4 Department of Psychiatry, Indiana University School of Medicine, Indianapolis, IN USA

**Keywords:** Human behaviour, Psychosis

## Abstract

Perinatal insults (e.g., obstetric complications, substance exposure) are increasing in prevalence and confer risk for psychotic-like experiences in offspring, contributing to a growing public health burden. Perinatal insults often co-occur, creating methodological challenges in understanding their impacts on psychosis-spectrum phenotypes. Data-driven approaches to organizing perinatal insults and testing their longitudinal effects on psychotic-like experiences in youth increases ecological validity and translational utility. Using data from 11,417 youth ages 9–14 across five years of the Adolescent Brain Cognitive Development (ABCD) Study, data-driven dimensions of perinatal insults were derived through exploratory factor analysis of thirty-one perinatal insults. Latent growth modeling tested the effect of perinatal insult dimensions on trajectories (baseline, rate-of-change, year-four severity) of distressing psychotic-like experiences. Six dimensions of perinatal insults were observed (substance exposure, obstetric complications, birth complications, postnatal challenges, parental age, medical needs). Substance exposure (β = 0.42, 95% CI [0.20, 0.63]), obstetric complications (β = 0.34, 95% CI [0.08, 0.61]), and parental age (β = 1.00, 95% CI [0.76, 1.22]) were associated with elevated baseline psychotic-like experiences. Perinatal insult dimensions were not associated with increasing rates-of-change in psychotic-like experiences. Medical needs (β = −0.12, 95% CI [−0.20, −0.05]) and parental age (β = −0.11, 95% CI [−0.18, −0.03]) were associated with steeper declines in psychotic-like experiences. Perinatal insult dimensions remained associated with elevated psychotic-like experiences at year-four. Data-driven dimensions of perinatal insults are associated with stably elevated psychotic-like experience trajectories across early adolescence. Given the role of psychotic-like experiences in later psychopathology and functioning, early identification of at-risk offspring is critical in reducing the public health burden of these exposures.

## Introduction

Perinatal insults like substance exposure, obstetric complications, and birth/delivery complications are increasing globally^1–4^ (~15% in the United States specifically in the last decade) further compounded by the COVID-19 pandemic^5^ and looming sociopolitical changes^6^. Exposure to perinatal insults is associated with psychopathology throughout development^7,8^, particularly psychosis-spectrum phenotypes^9–14^, imposing significant individual, familial, and economic burden^15,16^ and rendering this a major public health challenge. Difficulties modeling the heterogeneity of perinatal insults has limited their inclusion in early risk assessment, prevention, and intervention frameworks. This is likely because (1) there is currently no agreement on how to organize perinatal insults into a meaningful and useful framework and (2) it remains unclear how perinatal insult dimensions are associated with trajectories of psychotic-like experiences early in development.

Perinatal insults are typically modeled in two ways: specificity (i.e., testing one insult on an outcome^10,17^) and cumulative risk (i.e., testing the non-specific sum of insults on an outcome^10,18^). While numerous factors limit these approaches, a notable reality is that perinatal insults often co-occur within individuals^19,20^. For example, maternal hypertension is associated with prematurity^21^ and low birthweight^22^, whereas exposure to alcohol in utero is associated with concurrent cannabis and tobacco exposure^23^. This co-occurrence renders interpreting the effect of any one of these insults on psychopathology challenging. It can be difficult to parse whether an observed effect is a function of the measured insult or some other co-occurring but unmeasured exposure. Instead, perinatal insults likely aggregate into data-driven dimensions of conceptually and mechanistically similar environmental experiences with differential effects on psychopathology^24^. Data-driven dimensions of environmental risks are widely derived in studies of childhood insults, which demonstrate shared and unique effects of childhood insult dimensions on psychopathology^25–29^. Unfortunately, such an approach remains sparse in considerations of the perinatal period (though see ref. ^30^). These ecologically valid frameworks hold untapped value to perinatal insult conceptualization and interpretation.

Though specific and cumulative perinatal insults are associated with elevated psychosis phenotypes across development, the literature is overwhelmingly cross-sectional. For example, substance exposure and obstetric complications are risk factors for psychosis-spectrum phenomena independently in childhood^10^, adolescence^13^, and adulthood^9^. While informative, cross-sectional approaches neglect a fundamental principle: childhood through early adolescence represents a sensitive window of development wherein subclinical psychosis-spectrum phenotypes (e.g., psychotic-like experiences) often emerge^31,32^ and, in some, increase, conferring risk for transitioning to clinical high risk or frank psychosis^33,34^. Given the extant literature to date, it is unclear whether perinatal insults are associated with increasing psychosis symptoms across this period. Understanding how perinatal insult dimensions, which may serve as potentially modifiable etiological factors, influence trajectories of subclinical psychotic-like experiences throughout a window of development where such phenomena may first be assessed in clinical settings is critical to actionable translation.

Specificity and cumulative risk approaches in modeling perinatal insults have contributed to difficulty identifying mechanisms by which these insults confer risk for future elevations in psychotic-like experiences. While various mechanistic theories on how these risk factors influence psychopathology have gained support (e.g., that psychopathology develops from the cumulative impact of distinct “hits,” i.e., the two-hit hypothesis^35^; genetic mutation^36^), no one theory has dominated. Shared among extant theoretical frameworks is the potentially deleterious influence of perinatal insults on early neurodevelopment, which may in turn confer risk for maladaptive downstream outcomes. For example, some perinatal insults such as substance exposure and obstetric complications exert direct influences on neurodevelopment^37–40^. Others, like parental age and delivery type indirectly influence neurodevelopment^41,42^. As such, different dimensions of co-occurring perinatal insults may link to unique theoretical models and mechanisms, and ultimately distinct prevention and intervention efforts. To meaningfully test these models and mechanisms, it is necessary to first derive a framework through which modeling perinatal insults balances excessive splitting (i.e., specificity) and lumping (i.e., cumulative risk) of insults^43^.

The current longitudinal cohort study leveraged the Adolescent Brain Cognitive Development (ABCD) Study to address these limitations through two primary aims. First, perinatal insult dimensions were derived in a data-driven manner to generate a meaningful, ecologically valid framework by which to test longitudinal effects of perinatal insults. It was hypothesized that data-driven dimensions would largely reflect canonical categorizations of perinatal insults (e.g., obstetric complications, substance exposure) with certain insults being assimilated into dimensions into which they are not typically considered (e.g., unexpected pregnancy and substance exposure). Second, effects of perinatal insult dimensions on longitudinal trajectories of distressing psychotic-like experiences were estimated using annual data in youth from age 9 to 14. Dimensions were expected to have broad positive associations with baseline (age 9–10), rates-of-change, and year-four (age 13–14) severity across psychotic-like experiences.

## Methods

### Participants

All data are from the Adolescent Brain Cognitive Development (ABCD) study, an ongoing, longitudinal, population-based, cohort study of youth ages 9–10 at baseline (*N* = 11,868) and 13–14 (*N* = 4754, half of participants per ABCD data release schedule) at year-four. Data are collected from 21 research sites geographically distributed across the United States. Full recruitment details are documented elsewhere^44^; inclusion and exclusion criteria can be found in the supplementary material. Centralized institutional review board approval was obtained from the University of California, San Diego. Written consent/verbal assent was obtained from caregivers and youth. Analyses use data from all waves of the ABCD Study 5.1. Demographic characteristics can be found in Table 1.

**Table 1:** Demographic Characteristics and Psychopathology at Each ABCD Study Wave.

ABCD Study Wave [T0 (Baseline) – T4 (Year-Four Follow-Up)]					
	T0 (*n* = 11 417)	T1 (*n* = 10 809)	T2 (*n* = 10 577)	T3 (*n* = 9969)	T4 (*n* = 4596)
**Age** (y) *[Range]*	9.91 (0.62) [8–11]	10.92 (0.64) [9–12]	12.02 (0.67) [10–14]	12.91 (0.65) [11–15]	14.08 (0.68) [12–16]
**Sex**^**a**^ **assigned at birth** *Number (%)*					
Female	5455 (48)	5150 (48)	5024 (48)	4730 (48)	2181 (48)
Male	5916 (52)	5658 (52)	5552 (52)	5328 (52)	2415 (52)
**Race/Ethnicity**^**b**^ *Number (%)*					
White	6054 (53)	5875 (54)	5752 (54)	5490 (55)	2632 (57)
Black	1638 (14)	1467 (14)	1435 (14)	1252 (13)	471 (10)
Hispanic	2325 (20)	2146 (20)	2096 (20)	2009 (20)	949 (21)
Asian	215 (2)	204 (2)	194 (2)	185 (2)	89 (2)
Other^c^	1182 (10)	1114 (10)	1098 (10)	1031 (10)	455 (10)
***Psychotic-Like Experiences***					
	T0 (*n* = 11 406)^d^	T1 (*n* = 10 802)	T2 (*n* = 10 546)	T3 (*n* = 9943)	T4 (*n* = 4580)
Total (SD)	2.62 (3.54)	1.91 (3.16)	1.58 (2.81)	1.27 (2.45)	1.13 (2.31)
Distress Score (SD)	6.26 (10.54)	4.54 (9.20)	3.58 (7.75)	2.85 (6.49)	2.60 (6.52)
Family History^e^	1161 (10)	1097 (10)	1069 (10)	995 (10)	489 (11)
Number (%) [Median]	[3.5]	[3.5]	[3.5]	[3]	[2.75]

^a^*n* = 1 missing biological sex data.

^b^*n* = 3 missing race/ethnicity data for T0 and T1, *n* = 2 for T2 and T3.

^c^ “Other” category includes Native American/Alaska Native, Native Hawaiian or Other Pacific Islander, multiple and other (unspecified).

^d^*n* = 1 missing PLE data at T0.

^e^Family History item reflects “seeing visions or hearing voices or thinking people were spying on them or plotting against them”.

### Perinatal insult dimensions

Thirty-eight perinatal insults from the caregiver-reported Developmental History Questionnaire^45–47^ were originally identified for analyses. Insults spanned substance exposure, obstetric complications, birth and delivery complications, and various caregiver circumstances (e.g., nutrition, age at conception, unexpected pregnancy), with prevalence rates consistent with those observed in both cross-sectional and prospective studies (cf.^18^). Items endorsed by fewer than 1% of participants were removed prior to analyses to ameliorate potential model convergence, fit, and interpretation concerns [*n* = 4 items; proteinuria (0.47%), rubella (0.15%), convulsions (0.15%), blood transfusion (0.47%)]. Items correlated >0.90 were combined (*n* = 2 items) into one variable to attenuate multicollinearity. A final set of 31 perinatal insults were used for analyses. Full details on perinatal insults, including specific items, scoring, and endorsement/average are provided in Table S1.

### Psychopathology outcomes

Psychotic-like experiences (PLEs) were assayed with the youth-reported Prodromal Questionnaire-Brief Child Version (PQ-BC^48^). The PQ-BC is a 21-item questionnaire that assesses positive PLEs (e.g., perceptual abnormalities, mild delusional thoughts) over the past month and has been validated in the ABCD sample^48^, providing more range and specificity than psychosis-related subscales from other measures. Analyses used distress scores [0 (not endorsed), 1 (endorsed with no distress) or 2–6 (1+distress score), range: 0–126]; supplemental analyses used sum scores (range: 0–21). Internalizing and externalizing symptoms were assayed with the parent-reported Child Behavior Checklist (CBCL)^49^. The CBCL is a widely used psychopathology rating scale comprised of 119 total items. Each item is rated on a three-point scale ("not true," "somewhat true," or "very true"). Analyses used raw scores (32 internalizing items, range: 0–96; 27 externalizing items, range: 0–81). The PQ-BC and CBCL are administered annually beginning at the ABCD study baseline visit. Descriptive statistics can be found in Table 1.

### Statistical analyses

#### Overview

Analyses were conducted with R 4.2.0 and MPlus 8.11. To account for non-independence of observations, all models estimated cluster-robust standard errors with the sandwich estimator. Specifically, all models clustered data by family membership to account for siblings/multiple births and stratified data by research site. Additionally, analyses were weighted by poststratification weights provided by the ABCD Study to increase the representativeness of the sample. Together, this approach increases the validity of standard error estimates (thereby decreasing the potential for Type I error) and is consistent with previous work using the ABCD sample^50,51^.

#### Exploratory factor analysis & exploratory structural equation modelling

Thirty-one perinatal insults were included. Individuals missing >15% of perinatal insult data were removed from analyses (*n* = 446) (cf.^30^). For the remainder of youth, missing perinatal insults were imputed with an established imputation algorithm^52^. This non-parametric technique to estimating missing data utilizes a random forest imputation algorithm using the observed values to predict missing values, is uniquely suited to impute mixed categorical and continuous datasets, and outperforms other imputation models and case/variable deletion^52,53^.

Perinatal insult dimensions were derived using exploratory factor analysis (EFA) sensitive to variable type (e.g., continuous vs. dichotomous). Models with 1–10 factors were estimated using the weighted least squares mean and variance adjusted (WLSMV) estimator. The best-fitting model was selected based on a combination of (1) visual analysis of the Scree plot, (2) model fit statistics [Root Mean Squared Error of Approximation (RMSEA <0.05), Comparative Fit Index (CFI >0.95), Tucker-Lewis Index (TLI >0.95), Standardized Root Mean Squared Residual (SRMR <0.08)]^54^, and (3) conceptual interpretability. Item factor loadings above 0.30 were considered meaningful.

To obtain individual-level factor scores (i.e., scores on perinatal insult dimensions), exploratory structural equation modeling (ESEM)^55^ was conducted specifying the best-fitting number of factors from the EFA. Items that did not meaningfully load on to any factor (*n* = 5) were removed prior to ESEM. Factor scores were standardized prior to analyses. Full detail regarding the usefulness of an EFA plus ESEM approach for modeling early life risk factors are in supplemental materials.

#### Longitudinal modeling

Latent growth modeling (LGM)^56^ was used to test longitudinal associations between perinatal insult dimensions and trajectories of psychotic-like experiences. LGM is a structural equation modeling technique that estimates between-person differences in within-person change over time. First, unconditional LGMs established whole-sample trajectories for psychotic-like experiences. Second, perinatal insult dimension scores were entered as predictors of baseline (intercept) and rate-of-change (slope) for PLEs. Third, a model specifying the intercept at year four was estimated to evaluate perinatal insult dimension effects on PLEs at the final time-point. Specifying the intercept at the final timepoint, used to determine year-four symptom severity, does not alter model fit. Including both an intercept and slope factor simultaneously controls for baseline and rate-of-change in symptoms. LGMs used the robust maximum likelihood estimator. Model fit was evaluated via RMSEA (<0.05), CFI (>0.95), TLI (>0.95), and SRMR (<0.08)^54^. Missing psychopathology values were estimated with full information maximum likelihood (FIML) estimation under the robust maximum likelihood estimator. FIML outperforms other approaches to addressing missing data, including listwise and pairwise deletion, resulting in less biased parameter estimates^57^. All longitudinal models were adjusted for baseline age, race and ethnicity, biological sex, and family history of psychopathology. Results reported here reflect raw psychotic-like experience distress scores. To probe whether perinatal insult dimension conferred similar risk for other domains of psychopathology, we followed the same series of analytic steps with internalizing and externalizing psychopathology (Table S2 and Table S3). Including other demographic covariates (Area Deprivation Index, family income, parental self-reported psychopathology) and running models on standardized psychopathology scores did not substantively change the results (Table S4 and Table S5).

## Results

Demographic details are provided in Table 1. Youth were 9.90 (SD = 0.62) at baseline and 14.08 (SD = 0.68) at year four. Forty-eight percent were female, 52% were male and majority (53%) were non-Hispanic White.

### Perinatal insult dimensions

**Fig. 1:**
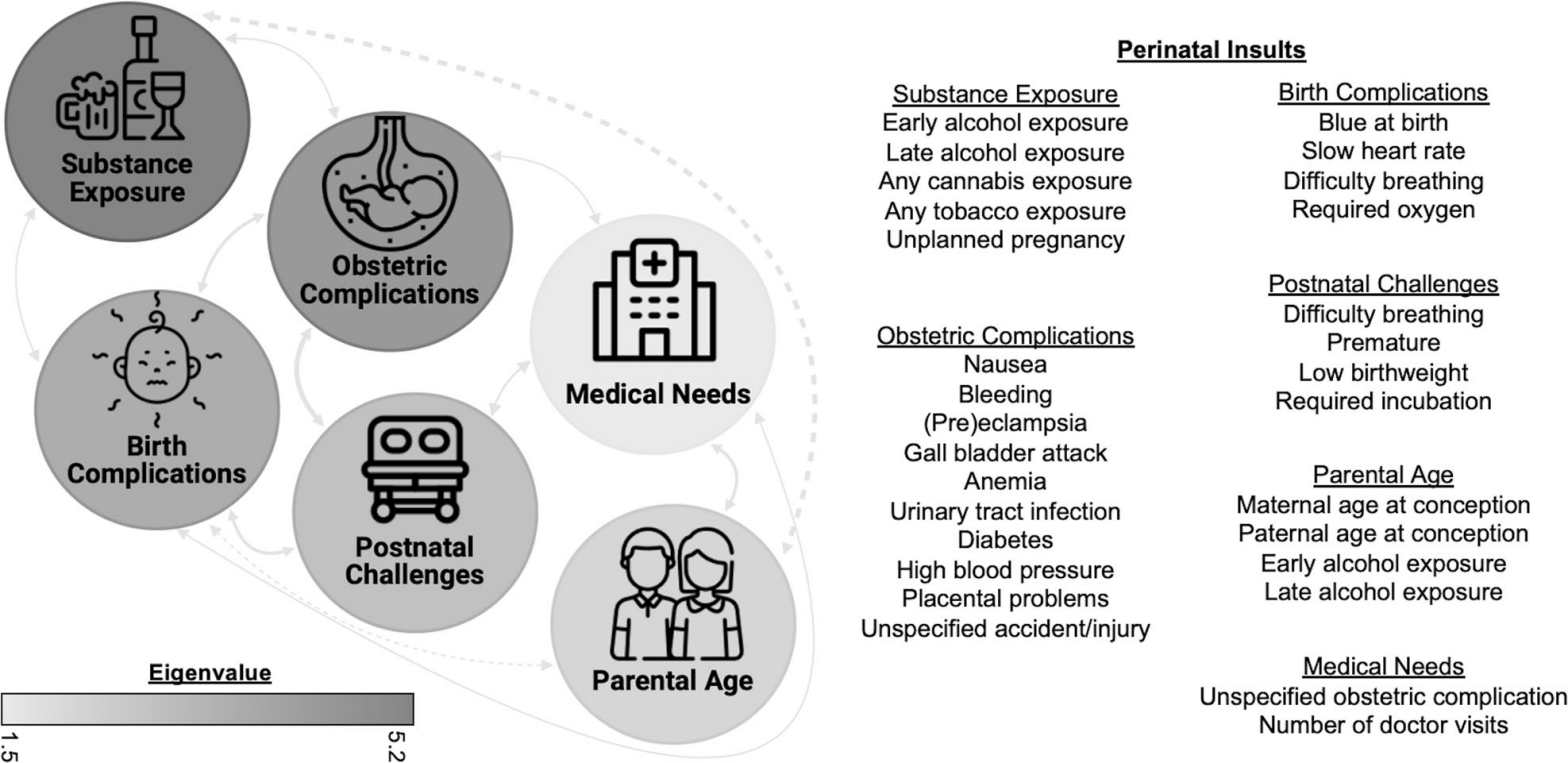
Results from exploratory factor analysis on perinatal insults. Data-driven dimensions of perinatal insults obtained through exploratory factor analysis. The density of shading corresponds to the eigenvalue for the dimensions (darker=higher). Solid arrows indicate positive correlations between dimensions; dashed arrows indicate negative correlations between dimensions. Specific items, their loadings, eigenvalues for each dimension, and correlations between dimensions are presented in Table S1, Tables S6–S8, and Figure S1.

A 6-factor solution for perinatal insult dimensions best explained the co-occurrence of PIs (Fig. 1) (X^2^(294) = 158.87, *p* <0.001; RMSEA = 0.02, CFI = 0.96, TLI = 0.94, SRMR = 0.06). These dimensions reflected (1) substance exposure and unexpected pregnancy (“substance exposure”), (2) obstetric complications (“obstetric complications”), (3) birth complications (“birth complications”), where higher scores indicate more exposure within the dimension; (4) postnatal challenges (“postnatal challenges”), where higher scores reflect lower birthweight, earlier birth, and longer incubation time; (5) parental age and alcohol use (“parental age”), with higher scores representing younger parental age at conception; and (6) medical needs (“medical needs”), where higher scores indicate increasing number of medical visits and unspecified obstetric complications. Notably, alcohol exposure cross-loaded onto substance exposure and parental age dimensions. Requiring oxygen at birth cross-loaded onto birth complications and postnatal challenges dimensions. Item loadings (Table S6), model fit statistics for other EFA estimations (Table S7), relationships between perinatal insult dimensions (Table S8) and the EFA scree plot (Figure S1) can be found in the Supplemental Material.

### Psychotic-like experiences

**Fig. 2:**
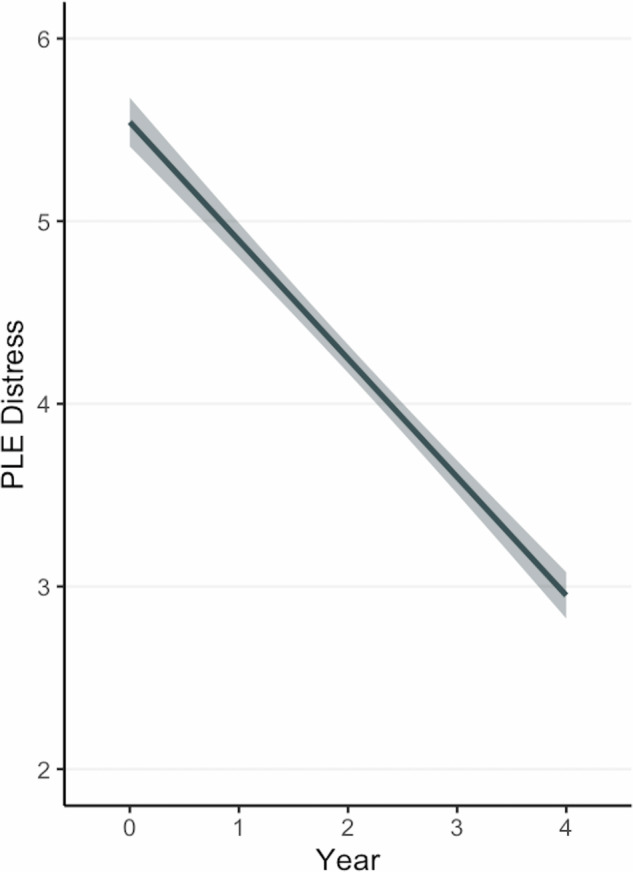
Full sample trajectories of distressing psychotic-like experiences across time. Longitduinal trajectory of Psychotic-Like Experiences distress scores at the sample-level across ABCD Study waves. Mean age at Year 0 = 9.91-years-old; mean age at Year 4 = 14.08-years-old.

**Fig. 3:**
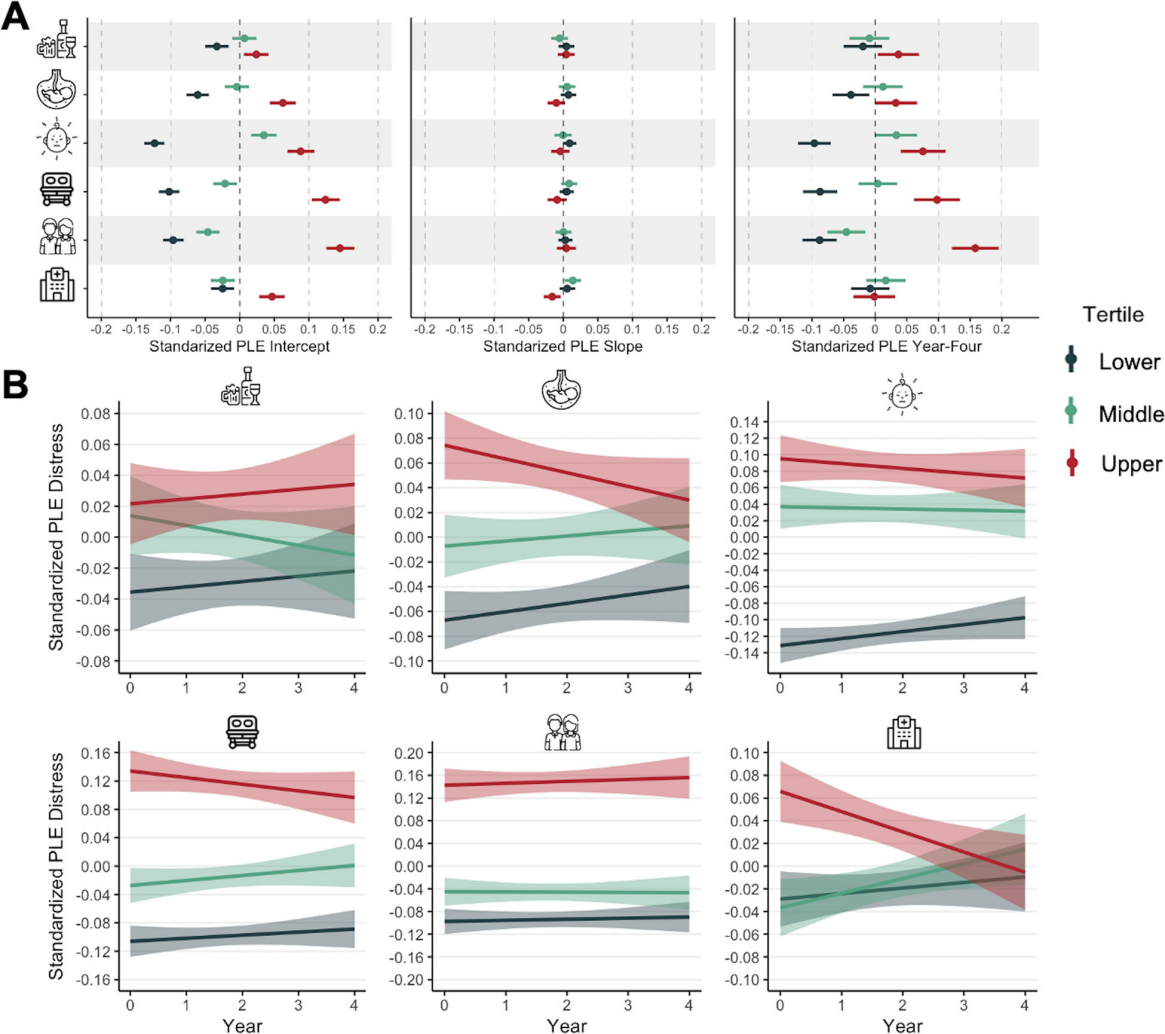
Associations between dimensions of perinatal insults and trajectories of psychotic-like experiences. **A** Associations between perinatal insult dimensions and baseline, slope, and year-four severity of Psychotic-Like Experiences distress scores. **B** Linear trajectories of Psychotic-Like Experience distress scores by perinatal insult dimension. The sample was divided into tertiles (bottom, middle, and top thirds) for each dimension of perinatal insult for visualization purposes only. For reported analyses, perinatal insult dimension scores were treated as continuous variables. Point estimates and 95% confidence intervals (visualized here) for each group-by-dimension figure and corresponding line graphs in are presented in Supplemental Tables 11–16.

On average, PLE distress decreased over time, with significant variability in intercept, slope, and year-four severity (Fig. 2, Table 2). Three perinatal insult dimensions were associated with greater distressing PLEs at baseline (age 9–10). Parental age had the largest effect (β = 1.00, 95% CI [0.76, 1.22]), followed by substance exposure (β = 0.42, 95% CI [0.20, 0.63]) and obstetric complications (β = 0.34, 95% CI [0.08, 0.61]). Medical needs and parental age dimensions were associated with distressing PLE slopes. Specifically, the medical needs dimension was associated with the steepest declines in PLE distress across time (β = −0.12, 95% CI [−0.20, −0.05]) and lower PLE distress at year-four (age 13–14) (β = −0.25, 95% CI [−0.42, −0.07]). That is, more frequent physician visits and unspecified obstetric complications were associated with faster declines in PLE distress across time that remained lower at the final timepoint. Similarly, the parental age dimension was negatively associated with the slope of PLE distress (β = −0.11, 95% CI [−0.18, −0.03]), such that younger age at conception was associated with faster declines in distressing PLEs across time. Of the dimensions associated with elevated baseline PLE severity, only the obstetric complications dimension was not associated with year-four PLE severity (age 13–14; Fig. 3A and B). Parental age had the largest effect at year-four (β = 0.58, 95% CI [0.39, 0.76]), followed by substance exposure (β = 0.28, 95% CI [0.10, 0.47]). Interestingly, the postnatal challenges dimension was only associated with year-four PLEs (β = 0.22, 95% CI [0.01–0.42]). Results were largely similar when evaluating trajectories of PLE sum scores (note, the parental age dimension association with slope became trend-level, *p* = 0.06) (Table S9).

**Table 2:** Results from Latent Growth Models for Psychotic-Like Experiences.

	**Psychotic-Like Experience Distress** X^2^(40) = 197.37; RMSEA = 0.02; CFI = 0.97; TLI = 0.95; SRMR = 0.02			
	B [95% CI]		*P*	
Baseline	15.13 [11.82, 18.45]		<0.001	
Slope	−2.14 [−3.17, −1.11]		<0.001	
Year-Four	6.58 [3.96, 9.19]		<0.001	
Baseline Variance	7.20 [6.75, 7.63]		<0.001	
Slope Variance	1.73 [1.52, 1.92]		<0.001	
Year-Four Variance	5.02 [4.50, 5.49]		<0.001	
	**Psychotic-Like Experiences**			
	**Baseline**	**Slope**		**Year-Four**
Substance Exposure	0.42*** [0.20, 0.63]	−0.33 [−0.10, 0.03]		0.28*** [0.10, 0.47]
Obstetric Complications	0.34** [0.08, 0.61]	−0.06 [−0.14, 0.02]		0.09 [−0.12, 0.29]
Birth Complications	0.08 [−0.13, 0.29]	0.01 [−0.06, 0.08]		0.13 [−0.06, 0.32]
Postnatal Challenges	0.15 [−0.10, 0.40]	0.02 [−0.06, 0.10]		0.22* [0.01, 0.42]
Parental Age	1.00*** [0.76, 1.22]	−0.11** [−0.18, −0.03]		0.58*** [0.39, 0.76]
Medical Needs	0.25 [−0.03, 0.53]	−0.12** [−0.20, −0.05]		−0.25** [−0.42, −0.07]

***p < 0.001, **p < 0.01, *p < 0.05.

### Internalizing and externalizing symptoms

On average, internalizing symptoms remained relatively stable across time (Figure S2a, see Table S2 for full model results). Obstetric complications (β = 0.54, 95% CI [0.35, 0.72]), parental age (β = 0.37, 95% CI [0.24, 0.50]), substance exposure (β = 0.35, 95% CI [0.23, 0.48]), and birth complications (β = 0.26, 95% CI [0.11, 0.42]) dimensions were associated with elevated internalizing symptoms at baseline (age 9–10). Similar to PLEs, parental age was negatively associated with the slope of internalizing symptoms (β = −0.10, 95% CI [−0.14, −0.06]). Substance exposure (β = 0.47, 95% CI [0.31, 0.64]) and obstetric complications (β = 0.45, 95% CI [0.25, 0.64]) dimensions remained associated with elevated symptoms at year-four (age 13-14); birth complications and parental age did not (Figure S3).

On average, externalizing symptoms modestly, but insignificantly, decreased across time (Figure S2b, see Table S2 for full model results). Parental age (β = 0.70, 95% CI [0.56, 0.83]), substance exposure (β = 0.64, 95% CI [0.50, 0.78]), obstetric complications (β = 0.43, 95% CI [0.24, 0.61]), and birth complications (β = 0.25, 95% CI [0.10, 0.40]) dimensions were associated with elevated externalizing symptoms at baseline (age 9-10). Parental age (β = −0.09, 95% CI [−0.12, −0.05]) and birth complications (β = −0.05, 95% CI [−0.09, −0.02]) dimensions were associated with faster declines in trajectories of externalizing symptoms. Except for birth complications, these dimensions continued to be associated with elevated year-four (age 13-14) symptom severity, with substance exposure demonstrating the largest effect (β = 0.60, 95% CI [0.44, 0.76]) (Figure S4).

## Discussion

Perinatal insults are robust predictors of psychosis-spectrum phenotypes throughout development. However, existing approaches to modeling perinatal insults have been limited in their conceptual and translational utility as they do not account for the co-occurrence of insults within individuals and have been largely limited to cross-sectional outcomes. The current work leveraged a data-driven approach with a national sample to organize perinatal insults into a meaningful, ecologically valid framework. The present analyses converge on a key finding – starting at age nine, ecologically-valid dimensions of perinatal insults are associated with elevations in psychotic-like experiences that remain stable throughout late childhood into early adolescence.

A best-fitting six-dimension solution organized the co-occurrence of specific perinatal insults into a novel framework, distilling into (1) Substance Exposure, (2) Obstetric Complications, (3) Birth Complications, (4) Postnatal Challenges, (5) Parental Age, and (6) Medical Needs. While these dimensions largely reflect canonical categorizations of perinatal insults, several notable differences emerged. First, unexpected pregnancy loaded onto the substance exposure factor, consistent with results from a network approach to dimension-reduction of perinatal insults^30^. Indeed, this may reflect accidental substance use stemming from not expecting to become pregnant as opposed to clinically significant substance misuse throughout pregnancy. Second, alcohol exposure loaded on two factors – substance exposure and parental age. Given alcohol use loads substantially stronger onto the substance exposure factor, the smaller loading of alcohol exposure on parental age is potentially capturing normative age-related changes in alcohol use^58^. Finally, requiring oxygen at birth loaded onto both birth complications and postnatal challenges. Taken together, our analyses provide evidence that myriad perinatal insults can be organized into ecologically valid dimensions reflecting the co-occurrence of insults. These dimensions are more nuanced than, and extend beyond, general cumulative risk scores that have been overutilized in the extant literature. Utilizing this organizational framework for mechanistic and applied research could afford more robust and meaningful inferences regarding perinatal insult effects.

Perinatal insult dimensions exhibited broad associations with baseline psychotic-like experiences. Specifically, substance exposure, obstetric complications, and parental age were associated with elevated distressing psychotic-like experiences at baseline. No perinatal insult dimension was associated with fewer symptoms at baseline. Critically, these dimensions largely remained associated with elevated symptoms at the year-four follow-up. Multiple factors may contribute to the stability of elevated psychotic-like experience severity across time. First, healthcare systems may be doing a poor job at identifying offspring at greatest risk for future difficulties^59^. Understanding the developmental mechanisms through which perinatal insults confer risk for future psychopathology (e.g., alterations to fundamental developmental processes^60,61^) may mitigate this. Second, the persistence of perinatal insult impact may not be readily appreciated, such that current interventions are not tailored towards addressing underlying causes soon enough^60^.

However, compared to baseline, at year-four the obstetric complications dimension was no longer associated with PLEs distress. Notably, despite the medical needs dimension not being associated with baseline psychotic-like experiences, higher scores were associated with *lower* psychotic-like experience severity at year-four. It is interesting that the postnatal challenges dimension – comprised in part of birthweight and prematurity – was not associated with any outcome, contrary to existing research^62,63^. This may be a function of sampling, given that extremely low birthweight (<1200 grams) and extreme prematurity (<28 weeks gestational age) are ABCD exclusion criteria. Additionally, statistical limitations, including regression to the mean, measurement error in psychopathology rating scales, and reduction in rating scale “novelty” over time may underly these reductions.

Contrary to hypotheses, no perinatal insult dimension was associated with increasing psychotic-like experiences throughout adolescence. Perhaps counterintuitively, when relationships between PI dimensions and slopes were observed, they indicated faster decreases in symptoms across ages 9–14. Specifically, the medical needs factor was associated with greater rate of decline and lower year-four PLE severity. This dimension encapsulates two perinatal risk factors – number of physician visits and unspecified obstetric complications – that may index higher utilization of healthcare systems^64,65^. That is, youth higher on this dimension may have more access (i.e., well-resourced or due to need) to systems that are equipped to detect PLEs early and intervene accordingly. Future studies focused on healthcare utilization may better clarify the complex factors influencing access to care. Similar accelerated declines were seen between the parental age dimension psychotic-like experiences, where higher factor scores indicate younger ages at conception. Younger parents have more external support (e.g., familial involvement in child rearing, multiple streams of income^66,67^; or are in more frequent contact with support systems as a function of risks associated with earlier conception^68^, that may buffer against the impact of parental age on early psychotic-like experiences. Thus, access to care, healthcare contact and utilization, and familial support appear to be effective in attenuating risk for early PLE elevations above and beyond what may be a normative decrease in PLEs observed in the sample.

While perinatal insults have primarily been studied as a risk factor for psychosis-spectrum phenotypes^9^, the current findings add to the growing literature highlighting perinatal insults as a transdiagnostic psychopathology risk factor^7,8^. In the present work, perinatal insult dimension effects were not specific to elevated trajectories of psychotic-like experiences but demonstrated unique and shared associations with stably elevated trajectories of internalizing and externalizing symptoms (Table S10). This is unsurprising given the high comorbidity and phenomenological overlap of early psychotic-like experiences with other psychopathologies, especially in early development^69,70^. Similar patterns across psychopathologies may reflect similar etiopathological mechanisms of perinatal insults (e.g., disrupted neurodevelopment^71^, increased inflammation^72^) as clinical phenomenology emerges and differentiates (i.e., prodromal syndromes, frank psychosis). Conversely, it may be that perinatal insult dimensions exert effects on psychopathology at a broader level (e.g., the p-factor^73^) that is captured in analyses of specific domains of psychopathology. However, it should be considered that differences between perinatal insult dimensions and trajectories of different psychopathology domains may be, in part, a function of different reporters (youth-reported PLEs, caregiver-reported internalizing and externalizing symptoms).

The current results provide evidence that perinatal insult dimensions affect trajectories of psychotic-like experiences in varying ways, suggesting these insults may operate through unique paths or mechanisms. Moving these findings forward, accounting for the co-occurrence of perinatal insults may aid in supporting or clarifying theoretical models proposing how these potentially modifiable environmental factors uniquely confer risk for psychotic-like experiences. For example, one way in which the parental age dimension may influence offspring psychotic-like experiences is through de-novo mutations, which have been shown to be a risk factor for psychosis-spectrum phenotypes^74,75^. However, the parental age dimension may also confer risk through psychosocial mechanisms (e.g., financial stability, perinatal health literacy) discussed above. Accordingly, for individuals higher on this dimension, advancements in gene therapy or increasing home support may be beneficial in attenuating risk. The substance exposure and obstetric complications dimensions may be more strongly associated with oxidative stress mechanisms. Indeed, substance exposure and obstetric complications are associated with elevated oxidative stress markers and associated cellular damage^76,77^, which may increase risk for psychopathology in offspring^78,79^. Efforts to reduce maternal substance use and increase maternal physical health may support a decrease in offspring psychotic-like experiences. Alternatively, certain substances (e.g., alcohol, cannabis) have targeted effects on neurodevelopment, including brain regions implicated in psychosis risk, which may provide a more unifying hypothesis than studying these brain regions in isolation^37,80^. Finally, it is possible that perinatal insult dimensions in-and-of themselves confer a small degree of risk for later psychotic-like experiences and instead, in non-genetically predisposed youth, serve as a first “hit” that disrupts early development, with behavioral changes not observed until a second, later, “hit” (e.g., trauma, parental psychopathology, substance use) triggers the system^35^. Alternatively, perinatal insults may be the second “hit,” with genetic proneness (or a co-occurring dimension conferring genetic risk) being the first “hit.” Future studies testing the two-hit hypothesis with gene-by-environment interactions and/or dimensions of perinatal and childhood insults may be informative in allocating resources, prevention, and intervention efforts. Critically, perinatal insults disrupt global structural and functional neurodevelopment (e.g., see refs. ^37,81,82^), suggesting disrupted neurodevelopment as a shared mechanism. Future work is required to test how perinatal insult dimensions derived here may map onto such theoretical models, influence neurodevelopment and, ultimately, affect functional outcomes that confer risk for psychosis-spectrum phenotypes (e.g., motor, social, and cognitive development).

Considered holistically, in perinatal insult-exposed youth, early identification combined with structured support systems may be critical in offsetting future stable psychotic-like experience elevations a decade later. Though the mechanisms through which dimensions of perinatal insults are associated with these elevations in psychotic-like experiences prior to and throughout adolescence warrant future research, the perinatal period is a window of time where caregivers are in frequent contact with healthcare systems and may be ripe for identification efforts and implementation of perinatal stepped care models^60^.

Several limitations and future directions are worth addressing. First, all perinatal insult data was retrospectively reported 9–10 years after pregnancy. While this may have introduced a non-negligible degree of recall bias, previous longitudinal research has demonstrated that most perinatal risk factors (including binary metrics of substance use, obstetric, and birth complications) can be reliably recalled a decade post-partum compared to medical records at birth^83–85^, though some insults (e.g., continuous metrics of substance use, unexpected pregnancy) are more variable^84,86^. Second, the current approach to testing trajectories of psychopathology does not account for potential latent trajectory groups within the ABCD sample (i.e., subgroups of individuals characterized by different patterns of PLE trajectories)^87,88^. Future research testing whether perinatal insult dimensions are differentially associated with trajectory subgroups may be informative in further parsing psychopathology risk. Third, while the relative timing of some insults is known, specific information on the temporality of perinatal insults is not available. For example, for some insults (e.g., certain substances or obstetric complications), when and how long they occurred may shift their placement in or alter the structure of the dimensional model. Fourth, certain potent perinatal insults are not captured by the ABCD study that may give more depth to perinatal insult dimensions, such as caregiver stress^89^, inflammation during pregnancy^90^, and perinatal socioeconomic status^91^. Replicating and extending findings in other longitudinal datasets with perinatal data collection will be critical in refining this framework. Fifth, the removal of four perinatal insults with less than 1% endorsement may have excluded rare, but highly potent risk factors for psychopathology (e.g., rubella, convulsions)^92,93^. Longitudinal research with help-seeking birthing persons may increase the representation of these insults. Sixth, future work would benefit from systematically testing interactions among perinatal insult dimensions, as such interactions may suggest synergistic or buffering effects on trajectories of psychotic-like experiences. Finally, these results should be considered in the context of the United States. Future research will benefit from testing whether these perinatal dimensions and their effects on psychopathology are similar cross-culturally.

Increasing rates of perinatal insults as a function of global sociopolitical changes necessitates a refined understanding of how these exposures are organized and confer risk for psychopathology longitudinally. The current study demonstrates that perinatal insults can be meaningfully organized into a dimensional framework that accounts for their co-occurrence, extending beyond widely used but limited approaches. Dimensions of perinatal insults exhibited unique and shared associations with stable elevations across multiple domains of psychopathology during a highly developmentally sensitive window. Early and frequent healthcare contact and utilization, as well as familial support, may be efficacious in attenuating risk for early PLE elevations. Efforts to understand the mechanisms by which these insults result in psychopathology elevations by age 9–10 with the use of prospective birth cohorts (e.g., Healthy Brain and Child Development, Avon Longitudinal Study of Parents and Children) and increasing availability of early identification and support services to expectant and new caregivers holds untapped potential in reducing the public health burden of these exposures.

## Supplementary information

Supplemental Material

## Data Availability

Adolescent Brain Cognitive Development (ABCD) Study data are publicly-available through the National Data Archive.
