# Publisher Correction: Perinatal insult dimensions and developmental trajectories of psychotic-like experiences

**DOI:** 10.1038/s41537-025-00691-1

**Published:** 2025-11-20

**Authors:** Eric R. Larson, Nicole R. Karcher, Alexandra B. Moussa-Tooks

**Affiliations:** 1 Department of Psychological & Brain Sciences, Indiana University, Bloomington, IN USA; 2 Program in Neuroscience, Indiana University, Bloomington, IN USA; 3 Department of Psychiatry, Washington University School of Medicine, St. Louis, MO USA; 4 Department of Psychiatry, Indiana University School of Medicine, Indianapolis, IN USA

**Keywords:** Human behaviour, Psychosis

Correction to: *Schizophrenia* 10.1038/s41537-025-00662-6, published online 25 August 2025

In the pdf version on this article, the table heading “Year-Four” and its corresponding entries in Table 2 were incorrectly merged with the first column. They should have appeared as a separate column at the end of the table. The original article has been corrected.

