# Supplementary material for: Perinatal insult dimensions and developmental trajectories of psychotic-like experiences
Source: Schizophrenia (Heidelb). 2025 Aug 25;11(1):115. doi: 10.1038/s41537-025-00662-6 (PMC12379231; doi:10.1038/s41537-025-00662-6)
Supplement: Supplementary file 1 — Supplemental Material [file 41537_2025_662_MOESM1_ESM.docx]

**SUPPLEMENTARY MATERIALS**

**Perinatal Insults Dimensions and Developmental Trajectories of Psychotic-Like Experiences**

Eric R Larson^1,2,*^, Nicole R. Karcher^3^, & Alexandra B Moussa-Tooks^1,2,4^

1. Department of Psychological & Brain Sciences, Indiana University, Bloomington, IN, US
2. Program in Neuroscience, Indiana University, Bloomington, IN, US
3. Department of Psychiatry, Washington University School of Medicine, St. Louis, MO, US
4. Department of Psychiatry, Indiana University School of Medicine, Indianapolis, IN, US

Supplemental Methods p. 3

Supplemental Table 1: Perinatal Insults p. 6

Supplemental Table 2: Internalizing and Externalizing Symptoms Across Waves p. 8

Supplemental Table 3: Longitudinal Effects of Perinatal Insult Dimensions on Internalizing and Externalizing Symptom Trajectories p. 9

Supplemental Table 4: Results with Additional Covariates p. 10

Supplemental Table 5: Fully Standardized Results p. 11

Supplemental Table 6: Perinatal Insult Factor Loadings p. 12

Supplemental Table 7: Model Fit Statistics for Exploratory Factor Analyses p. 13

Supplemental Table 8: Correlations Between Perinatal Insult Dimensions p. 14

Supplemental Table 9: Longitudinal Effects of Perinatal Insult Dimensions on PLE Sum Score Trajectories p. 15

Supplemental Table 10: Comparisons of Perinatal Insult Effect Size Magnitude between PLEs, Internalizing, and Externalizing Symptoms p. 16

Supplemental Tables 11-16: Perinatal Insult Dimension Tertiles and Psychotic-Like Experiences at Baseline, Slope, and Year-Four p. 17

Supplemental Tables 17-22: Perinatal Insult Dimension Tertiles and Internalizing

Symptoms at Baseline, Slope, and Year-Four p. 19

Supplemental Tables 23-28: Perinatal Insult Dimension Tertiles and Externalizing Symptoms at Baseline, Slope, and Year-Four p. 21

Supplemental Figure 1: Scree Plot p. 23

Supplemental Figure 2: Sample-Level Trajectories of Internalizing and Externalizing Symptoms p. 24

Supplemental Figure 3: Perinatal Insult Dimension Tertiles and Internalizing Symptom Trajectories p. 25

Supplemental Table 4: Perinatal Insult Dimension Tertiles and Externalizing Symptom Trajectories p. 26

References p. 27

**Supplemental Methods**

*Adolescent Brain Cognitive Development (ABCD) Study Inclusion and Exclusion Criteria*

Recruitment for the ABCD study occurs at 21 regionally-distributed sites throughout the United States. A full list of ABCD study sites can be found at <https://abcdstudy.org/study-sites/>. Exclusion criteria for the ABCD study include: MRI contraindication, history of traumatic brain injury, neurological disorder, current diagnosis of schizophrenia, intellectual disability, autism spectrum disorder, and/or alcohol or substance use disorder, gestational age less than 38 weeks, birthweight less than 1200 grams, and not fluent in English. More details on ABCD study design, recruitment, and assessments can be found elsewhere.^1–5^

*Family History of Psychopathology*

For each dimension of psychopathology separately, family history of the dimension was computed using the Family History Assessment Module Screener^6^ which assesses the presence of psychopathology across family members. For PLEs, the item “seeing visions or hearing voices or thinking people were spying on them or plotting against them” was used. For internalizing, the items “suffering from depression, that is, having felt so low for a period of at least two weeks that they hardly ate or slept or couldn't work or do whatever they usually do” and “other problems with their nerves, or had a nervous breakdown” were used. For externalizing, “been the kind of person who never holds a job for long, or gets into fights, or gets into trouble with the police from time to time, or had any trouble with the law as a child or an adult” was used to index family history.

**Appendix S2. Methodology Rationale**

*Data-Driven Derivation of Perinatal Insults Dimensions*

Within the extant research on early life insults, debate about how best to model the dimensionality of early life experiences is ongoing.^7^ While many approaches to deriving dimensions of adversity exist, two competing approaches are used that have different, but related, statistical and conceptual considerations – namely formative and reflective approaches.^8^ Both approaches attempt to derive “unobserved” or “unmeasured” latent constructs from characteristics of the data. Indeed, both approaches demonstrate that insults during childhood can be aggregated into meaningful dimensions, and these dimensions have shared and unique effects on psychopathology.^9–11^ The choice on which approach to use relies in large part on the research goals.

In formative models, variance in items (e.g., perinatal insults) is assumed to *cause* variance in some factor (e.g., dimension). In early life insult research, one formative model often used is Principal Components Analysis (PCA) (e.g.,^9,10,12^). That is, the “arrows” in the model point from the items to the latent constructs. The goal of this approach is to derive some unmeasured composites/factors that maximizes the total amount of variance explained by the items, both the shared and unique (including error-related variance.^13^ Typically, with formative modelling approaches, derived factors are mathematically orthogonal to each other – they are uncorrelated. In this sense, formative approaches are useful when the research goal is to operationalize dimensions of insults. In clinical psychological science, a prototypical example of formative models is socioeconomic status. It is theorized that changes in, say, household income, educational attainment, and occupational status cause changes in one’s broader socioeconomic status.

In reflective models, variance in items (e.g., perinatal insults) is assumed to be *caused by* variance in some factor (e.g., dimension). In early life insult research, one reflective model often used is Exploratory Factor Analysis (EFA) (e.g.,^10,11,14^). In these approaches, the “arrows” in the model point from the latent constructs to the items. The goal of this approach is to derive some unmeasured constructs/factors that account for shared variance (i.e., correlations) among items.^15^ Unlike formative models, which maximize total variance explained, reflective models permit an additional factor for each item that captures variance unique to the item itself and error-related variance. With reflective modelling approaches, derived factors are typically free to correlate with each other and item cross-loadings are allowed. In this sense, reflective approaches are useful when the research goal is to explain the co-occurrence of insults (cf.^16^). In clinical psychological science, a prototypical example of reflective models is broadband scales of psychopathology (e.g., internalizing symptoms). Internalizing symptoms may include items related to anxiety, depression, and somatic complaints (e.g., Child Behavior Checklist internalizing subscale). It is theorized that these items are correlated – they co-occur within individuals – and changes in any item are caused by changes in the broader construct.

The theoretical basis of this research is that perinatal insults often co-occur within individuals^17,18^ and that identifying patterns of co-occurrence while allowing dimensions to correlate will increase the ecological validity of perinatal insult assessment. Reflective models, and particularly EFA, are especially well-suited to address these research questions. If the goal of the present work was to operationalize constructs of perinatal insults through maximizing total explained variance, a formative approach may have been warranted.

*Obtaining Factor Scores*

Once a data-driven dimensional solution is obtained, it is necessary to compute factor scores to use these dimensions in predicting outcomes. There are various methods available to obtain factor scores once dimensions are obtained through EFA, two of which involve specifying the structure within a confirmatory factor analysis (CFA) framework or leveraging exploratory structural equation modelling (ESEM).

With a CFA approach, perinatal insults are specified to load onto one specific dimension, usually that in which the item loads most strongly. Of note, cross-loadings are constrained to zero. While this approach appreciates a parsimonious dimensional structure, it also tends to be overly restrictive and not consistent with the goals of deriving dimensions based on insult co-occurrence. As such, CFA-derived factor scores may not adequately reflect the complexity of perinatal insult co-occurrence, can lead to inaccurate factor scores by restricting cross-loadings, and can result in a poor fit to the data.

Instead, the goals of deriving factor scores via exploratory structural equation modelling (ESEM) are consistent with complexity of perinatal insult burden. ESEM is a confirmatory technique that merges methods from exploratory and confirmatory factor analysis.^19^ Unlike purely confirmatory techniques, exploratory structural equation modeling enables specification of item cross-loadings and factor correlations, while still providing conventional model fit statistics, resulting in less biased and more realistic model estimates. Thus, factor scores are more reflective of the underlying data-driven dimensional model.

| **Supplemental Table 1. Perinatal Insults Used in Current Study** | | | | | | | | |
| --- | --- | --- | --- | --- | --- | --- | --- | --- |
| **Item Content** | **ABCD Variable(s)** | **MPlus Code** | **Scoring** | | **% Missing (Pre-Imputation)** | | | **% Endorsement (dichotomous) or Median [Mean] (count/continuous)** |
| **Items Included into Dimension Reduction with Factor Loadings >0.30** | | | | | | | | |
| Alcohol exposure (before knowledge of pregnancy) | devhx_8_alcohol | alc_e | 0=No 1=Yes | | 3.85% | | | 25.49% |
| Tobacco exposure | devhx_8_tobacco; devhx_9_tobacco | tob | 0=No 1=Yes | | 0.56% | | | 13.44% |
| Cannabis exposure | devhx_8_marijuana; devhx_9_marijuana | can | 0=No 1=Yes | | 0.91% | | | 5.69% |
| Alcohol exposure (after knowledge of pregnancy) | devhx_9_alcohol | alc_l | 0=No 1=Yes | | 0.43% | | | 2.39% |
| Severe nausea and vomiting | devhx_10a3_p | nausea | 0=No 1=Yes | | 0.42% | | | 14.04% |
| Heavy bleeding | devhx_10b3_p | bleed | 0=No 1=Yes | | 0.10% | | | 4.48% |
| Pre-eclampsia, eclampsia, or toxemia | devhx_10c3_p | preecl | 0=No 1=Yes | | 0.48% | | | 7.55% |
| Severe gall bladder attack | devhx_10d3_p | gallbl | 0=No 1=Yes | | 0.09% | | | 1.12% |
| Severe anemia | devhx_10g3_p | anemia | 0=No 1=Yes | | 0.36% | | | 4.29% |
| Urinary tract infections | devhx_10h3_p | uti | 0=No 1=Yes | | 1.70% | | | 7.49% |
| Pregnancy-related diabetes | devhx_10i3_p | diabete | 0=No 1=Yes | | 0.36% | | | 7.03% |
| Pregnancy-related high blood pressure | devhx_10j3_p | highbp | 0=No 1=Yes | | 0.55% | | | 9.96% |
| Previa, abruptio, or other placental problems | devhx_10k3_p | placenta | 0=No 1=Yes | | 0.25% | | | 3.00% |
| Accident or injury requiring medical care | devhx_10l3_p | accident | 0=No 1=Yes | | 0.04% | | | 1.82% |
| Other conditions requiring medical care | devhx_10m3_p | otherob | 0=No 1=Yes | | 0.18% | | | 8.46% |
| Blue at birth | devhx_14a3_p | blue | 0=No 1=Yes | | 0.99% | | | 3.36% |
| Slow heartbeat | devhx_14b3_p | slowhr | 0=No 1=Yes | | 1.05% | | | 2.97% |
| Did not breathe at first | devhx_14c3_p | breathe | 0=No 1=Yes | | 0.79% | | | 4.76% |
| Required oxygen | devhx_14f3_p | oxygen | 0=No 1=Yes | | 0.83% | | | 9.92% |
| Unexpected pregnancy | devhx_6_p | unexpect | 0=No 1=Yes | | 0.69% | | | 38.17% |
| Premature birth | devhx_12_p | premat | 1=1 week –  13=12+ weeks | | 0.00% | | | 0 [0.92] |
| Birthweight | birth_wight_lbs; birth_weight_oz | bw | 0= ≥2500 grams  1= <2500g | | 8.60% | | | 15.45% |
| Maternal age at birth | devhx_3_p | matage | Age | | 0.92% | | | 30 [29.45] |
| Paternal age at birth | devhx_4_p | patage | Age | | 3.52% | | | 32 [31.77] |
| Number of physician visits during pregnancy | devhx_11_p | docvis | Number of visits | | 8.50% | | | 14 [15.66] |
| Days of incubation postnatally | devhx_15 | incub | Number of days | | 4.11% | | | 0 [1.21] |
| **Items Included in Dimension Reduction but with Factor Loadings <0.30** | | | | | | | | |
| Jaundice needing treatment | devhx_14e3_p | jaund | 0=No 1=Yes | | 0.71% | | | 16.52% |
| Rh Incompatibility | devhx_14h3_p | rh | 0=No 1=Yes | | 0.94% | | | 2.50% |
| Number of months child was breastfed | devhx_18_p | breastfd | Number of months | | 1.96% | | | 6 [7.86] |
| C-section | devhx_13_3_p | csec | 0=No 1=Yes | | 0.09% | | | 38.19% |
| Prenatal vitamin use | devhx_10 | previt | 0=Yes 1=No | | 1.58% | | | 4.35% |
| **Items not included in analyses due to low (<1%) endorsement in the entire sample** | | | | | | | | |
| Persistent proteinuria | devhx_10e3_p | N/A | | 0=No 1=Yes | | N/A | 0.48% | |
| Rubella (German measles) | devhx_10f3_p |  |  | 0=No 1=Yes | |  | 0.15% | |
| Convulsions | devhx_14d3_p |  |  | 0=No 1=Yes | |  | 0.15% | |
| Required blood transfusion | devhx_14g3_p |  |  | 0=No 1=Yes | |  | 0.47% | |

| **Supplemental Table 2. Internalizing and Externalizing Symptoms Across Waves** | | | | | |
| --- | --- | --- | --- | --- | --- |
| ABCD Study Wave [T0 (Baseline) – T4 (Year-Four Follow-Up)] | | | | | |
|  | T0 (n = 11 417) | T1 (n = 10 809) | T2 (n = 10 577) | T3 (n = 9969) | T4 (n = 4596) |
| **Age** (y) *[Range]* | 9.91 (0.62)  [8 – 11] | 10.92 (0.64)  [9 – 12] | 12.02 (0.67)  [10 – 14] | 12.91 (0.65)  [11 – 15] | 14.08 (0.68)  [12 – 16] |
| ***Internalizing*** | | | | | |
|  | T0 (n = 11 413) | T1 (n = 10 789) | T2(n = 10 504) | T3 (n = 9741) | T4 (n = 4521) |
| Raw Score | 5.00 (5.46) | 5.08 (5.52) | 4.91 (5.62) | 5.10 (5.91) | 5.08 (6.03) |
| Family History^a^  Number (%) [Median] | 6568 (58)  [12.5] | 6217 (58)  [12.5] | 6164 (58)  [12.5] | 5773 (58)  [12] | 2694 (58)  [11.5] |
| ***Externalizing*** | | | | | |
|  | T0 (n = 11 413) | T1 (n = 10 789) | T2 (n = 10 504) | T3 (n = 9741) | T4 (n = 4521) |
| Raw Score | 4.33 (5.69) | 4.06 (5.50) | 3.81 (5.43) | 3.78 (5.34) | 3.39 (5.07) |
| Family History^b^  Number (%) [Median] | 3347 (29)  [6] | 3158 (29)  [6] | 3093 (29)  [5.5] | 2860 (29)  [5] | 1332 (29)  [5] |
| ^a^ Family History item reflects “suffering from depression, that is, having felt so low for a period of at least two weeks that they hardly ate or slept or couldn't work or do whatever they usually do” or “other problems with their nerves, or had a nervous breakdown”  ^b^ Family History item reflects “been the kind of person who never holds a job for long, or gets into fights, or gets into trouble with the police from time to time, or had any trouble with the law as a child or an adult” | | | | | |

| **Supplemental Table 3: Longitudinal Effects of Perinatal Insult Dimensions on Internalizing and Externalizing Symptom Trajectories** | | | | | | | | |  |
| --- | --- | --- | --- | --- | --- | --- | --- | --- | --- |
|  | **Internalizing**  X^2^(40)=139.33; RMSEA=0.02; CFI=0.99; TLI=0.99; SRMR=0.01 | | | | **Externalizing**  X^2^(40)=148.40; RMSEA=0.02; CFI=0.99; TLI=0.99; SRMR=0.02 | | | | |
|  | B [95% CI] | | P | | B [95% CI] | | P | | |
| Baseline | 2.41 [0.65-4.18] | | <0.01 | | 4.16 [2.35-5.69] | | <0.001 | | |
| Slope | 0.11 [-0.48-0.70] | | 0.71 | | -0.41 [-0.93-0.11] | | 0.123 | | |
| Year-Four | 2.86 [0.59-5.12] | | 0.014 | | 2.53 [0.44-4.61] | | 0.105 | | |
| Intercept Variance | 4.70 [4.51-4.87] | | <0.001 | | 4.93 [4.73-5.12] | | <0.001 | | |
| Slope Variance | 1.08 [1.00-1.15] | | <0.001 | | 0.96 [0.87-1.05] | | <0.001 | | |
| Year-Four Variance | 5.50 [5.27-5.72] | | <0.001 | | 4.96 [4.70-5.21] | | <0.001 | | |
|  | **Internalizing** | | | | **Externalizing** | | | | |
|  | **Baseline** | **Slope** | | **Year-Four** | **Baseline** | **Slope** | | **Year-Four** | |
| Substance Exposure | 0.35***  [0.23-0.48] | 0.03  [-0.01-0.07] | | 0.47***  [0.31-0.64] | 0.64***  [0.50-0.78] | -0.10  [-0.05-0.03] | | 0.60***  [0.44-0.76] | |
| Obstetric Complications | 0.54***  [0.35-0.72] | -0.02  [-0.07-0.03] | | 0.45***  [0.25-0.64] | 0.43***  [0.24-0.61] | -0.03  [-0.07-0.02] | | 0.32***  [0.14-0.50] | |
| Birth Complications | 0.26***  [0.11-0.42] | -0.04  [-0.08-0.01] | | 0.12  [-0.06-0.30] | 0.25**  [0.10-0.40] | -0.05**  [-0.09- -0.02] | | 0.03  [-0.12-0.18] | |
| Postnatal Challenges | -0.08  [-0.23-0.07] | -0.01  [-0.06-0.03] | | -0.14  [-0.33-0.05] | 0.03  [-0.14-0.20] | -0.02  [-0.06-0.02] | | -0.04  [-0.22-0.15] | |
| Parental Age | 0.37***  [2] | -0.10***  [-0.14- -0.06] | | -0.03  [-0.19-0.15] | 0.70***  [0.56-0.83] | -0.09***  [-0.12- -0.05] | | 0.35***  [0.20-0.51] | |
| Medical Needs | -0.21*  [-0.41- -0.01] | -0.04  [-0.03-0.06] | | -0.15  [-0.34-0.03] | -0.10  -0.31-0.11] | 0.01  [-0.03-0.05] | | -0.06  [-0.23-0.10] | |
| *** p<0.001; ** p<0.01, * p<0.05 | | | | | | | | |  |

| **Supplemental Table 4: Results from Latent Growth Models Including Area Deprivation Index, Family Income, and Caregiver Psychopathology as Covariates** | | | | | | | | | | | | |
| --- | --- | --- | --- | --- | --- | --- | --- | --- | --- | --- | --- | --- |
|  | **PLE Distress**  X^2^(49)=185.62; RMSEA=0.02; CFI=0.97; TLI=0.95; SRMR=0.02 | | | | **Internalizing**  X^2^(49)=204.30; RMSEA=0.02; CFI=0.99; TLI=0.98; SRMR=0.02 | | | | **Externalizing**  X^2^(49)=195.13; RMSEA=0.02; CFI=0.99; TLI=0.98; SRMR=0.02 | | | |
|  | N = 9,694 | |  | | N = 9,694 | |  | | N = 9,694 | |  | |
|  | B [95% CI] | | P | | B [95% CI] | | P | | B [95% CI] | | P | |
| Baseline | 14.83 [10.98-18.67] | | <0.001 | | 0.27 [-1.46-2.00] | | 0.76 | | 3.03 [1.16-4.90] | | <0.01 | |
| Slope | -1.90 [-3.04- -0.77] | | <0.01 | | 0.12 [-0.54-0.79] | | 0.72 | | -0.55] [-1.15-0.06] | | 0.08 | |
| Year-Four | 7.21 [4.37-10.05] | | <0.001 | | 0.76 [-1.70-3.21] | | 0.55 | | 0.84 [-1.47-3.15] | | 0.48 | |
| Intercept Variance | 7.04 [6.54-7.51] | | <0.001 | | 3.88 [3.71-4.04] | | <0.001 | | 4.27 [4.09-4.43] | | <0.001 | |
| Slope Variance | 1.65 [1.42-1.86] | | <0.01 | | 1.06 [0.99-1.14] | | <0.001 | | 0.93 [0.82-1.02] | | <0.001 | |
| Year-Four Variance | 4.73 [4.16-5.23] | | <0.001 | | 5.15 [4.92-5.34] | | <0.001 | | 4.70 [4.45-4.94] | | <0.001 | |
|  | **Psychotic-Like Experiences** | | | | **Internalizing** | | | | **Externalizing** | | | |
|  | **Baseline** | **Slope** | | **Year-Four** | **Baseline** | **Slope** | | **Year-Four** | **Baseline** | **Slope** | | **Year-Four** |
| Substance Exposure | 0.30**  [0.07-0.54] | -0.05  [-0.13-0.02] | | 0.09  [-0.10-0.29] | -0.13*  [-0.25- -0.01] | 0.05*  [0.01-0.10] | | 0.08  [-0.09-0.25] | 0.22**  [0.09-0.37] | 0.03  [-0.01-0.07] | | 0.33***  [0.17-0.50] |
| Obstetric Complications | 0.37*  [0.08-0.65] | -0.06  [-0.15-0.02] | | 0.12  [-0.10-0.34] | 0.24**  [0.08-0.40] | 0.004  [-0.05-0.06] | | 0.26*  [0.05-0.46] | 0.18*  [0.01-0.35] | 0.01  [0.04-0.05] | | 0.21**  [0.03-0.39] |
| Birth Complications | 0.04  [-0.18-0.26] | 0.02  [-0.05-0.09] | | 0.12  [-0.08-0.32] | 0.12  [-0.02-0.26] | -0.01  [-0.06-0.04] | | 0.08  [-0.10-0.26] | 0.11  [-0.04-0.25] | -0.02  [-0.06-0.02] | | 0.02  [-0.14-0.18] |
| Postnatal Challenges | 0.003  [0.26-0.26] | 0.04  [-0.05-0.12] | | 0.15  [-0.08-0.38] | -0.08  [-0.22-0.05] | 0.02  [-0.03-0.06] | | -0.02  [-0.21-0.17] | -0.01  [0.16-0.14] | -0.003  [0.05-0.04] | | -0.03  [-0.21-0.16] |
| Parental Age | 0.54***  [0.27-0.80] | -0.06  [0.14-0.03] | | 0.31**  [0.11-0.51] | -0.04  [-0.17-0.10] | -0.05*  [-0.10- -0.004] | | -0.25*  [-0.43-0.06] | 0.18*  [0.03-0.32] | -0.03  [-0.08-.01] | | 0.05  [-0.13-0.22] |
| Medical Needs | 0.11  [-0.14-0.37] | -0.10**  [-0.17- -0.02] | | -0.27**  [-0.45- -0.09] | -0.10  [-0.28-0.08] | -0.02  [-0.06-0.03] | | -0.16  [-0.36-0.03] | -0.01  [-0.19-0.18] | -0.02  [-0.06-0.03] | | -0.07  [-0.23-0.10] |
| *** p<0.001; ** p<0.01, * p<0.05  Note: N = 1,721 missing either Area Deprivation Index, Family Income, or Caregiver Psychopathology. Area Deprivation Index is a geocoded metric ranked as a percentile based on the participant’s self-reported address at the baseline visit. Family income is a 10-level binned metric of the combined family income (range <$25,000->$200,000). Caregiver psychopathology was the total raw score derived from the Adult Self Report rating scale. | | | | | | | | | | | | |

| **Supplemental Table 5: Fully Standardized Latent Growth Model Results** | | | | | | | | | | | | |
| --- | --- | --- | --- | --- | --- | --- | --- | --- | --- | --- | --- | --- |
|  | **PLE Distress**  X^2^(40)=197.37; RMSEA=0.02; CFI=0.97; TLI=0.95; SRMR=0.02 | | | | **Internalizing**  X^2^(40)=139.33; RMSEA=0.02; CFI=0.99; TLI=0.99; SRMR=0.01 | | | | **Externalizing**  X^2^(40)=148.40; RMSEA=0.02; CFI=0.99; TLI=0.99; SRMR=0.02 | | | |
|  | B [95% CI] | | P | | B [95% CI] | | P | | B [95% CI] | | P | |
| Baseline | 2.04 [1.57-2.52] | | <0.001 | | 0.49 [0.13-0.86] | | <0.01 | | 0.80 [0.45-1.15] | | <0.001 | |
| Slope | -1.20 [-1.79- -0.61] | | <0.001 | | 0.10 [-0.43-0.63] | | 0.71 | | -0.42 [-0.94-0.11] | | 0.120 | |
| Year-Four | 1.26 [0.75-1.78] | | <0.001 | | 0.50 [0.10-0.90] | | 0.014 | | 0.49 [0.08-0.90] | | 0.02 | |
| Intercept Variance | 0.97 [0.96-0.98] | | <0.001 | | 0.96 [0.95-0.96] | | <0.001 | | 0.94 [0.93-0.95] | | <0.001 | |
| Slope Variance | 0.97 [0.96-0.98] | | <0.001 | | 0.97 [0.97-0.98] | | <0.001 | | 0.99 [0.98-0.99] | | <0.001 | |
| Year-Four Variance | 0.96 [0.95-0.97] | | <0.001 | | 0.96 [0.96-0.97] | | <0.001 | | 0.97 [0.96-0.98] | | <0.001 | |
|  | **Psychotic-Like Experiences** | | | | **Internalizing** | | | | **Externalizing** | | | |
|  | **Baseline** | **Slope** | | **Year-Four** | **Baseline** | **Slope** | | **Year-Four** | **Baseline** | **Slope** | | **Year-Four** |
| Substance Exposure | 0.06***  [0.03-0.09] | -0.02  [-0.06-0.02] | | 0.06**  [0.02-0.09] | 0.07***  [0.05-0.10] | 0.03  [-0.01-0.07] | | 0.08***  [0.06-0.11] | 0.12***  [0.10-0.15] | -0.01  [-0.05-0.03] | | 0.12***  [0.09-0.15] |
| Obstetric Complications | 0.05**  [0.01-0.08] | -0.04  [-0.08-0.01] | | 0.02  [-0.02-0.06] | 0.11***  [0.07-0.15] | -0.02  [-0.07-0.03] | | 0.08 ***  [0.04-0.11] | 0.08***  [0.05-0.12] | -0.03  [-0.07-0.02] | | 0.06***  [0.03-0.10] |
| Birth Complications | 0.01  [-0.02-0.04] | 0.01  [-0.03-0.05] | | 0.03*  [-0.01-0.06] | 0.05**  [0.02-0.08] | -0.03  [-0.07-0.01] | | 0.02  [-0.01-0.05] | 0.05**  [0.02-0.08] | -0.06  [-0.09- -0.02] | | 0.01  [0.02-0.04] |
| Postnatal Challenges | 0.02  [-0.01-0.05] | 0.01  [-0.04-0.05] | | 0.04  [0.003-0.08] | -0.02  [-0.05-0.01] | -0.01  [-0.05-0.03] | | -0.02  [-0.06-0.01] | 0.01  [-0.03-0.04] | -0.02**  [0.06-0.02] | | -0.01  [0.04-0.03] |
| Parental Age | 0.14***  [0.11-0.17] | -0.06**  [-0.10- -0.02] | | 0.11***  [0.08-0.15] | 0.08***  [0.05-0.10] | -0.09***  [-0.13—0.05] | | -0.004  [-0.03-0.03] | 0.14***  [0.11-0.16] | -0.09***  [-0.13- -0.05] | | 0.07***  [0.04-0.10] |
| Medical Needs | 0.34  [-0.004-0.07] | -0.07**  [-0.12- -0.03] | | -0.05**  [-0.08- -0.02] | -0.04*  [-0.09- -0.001] | 0.01  [-0.03-0.06] | | -0.03  [-0.06-0.01] | -0.02  [-0.06-0.02] | 0.01  [-0.03-0.05] | | -0.01  [-0.05-0.02] |
| *** p<0.001; ** p<0.01, * p<0.05 | | | | | | | | | | | | |

| **Supplemental Table 6. Perinatal Insult Factor Loadings for the 6-dimension solution** | | | | | | |
| --- | --- | --- | --- | --- | --- | --- |
|  | Factor 1 | Factor 2 | Factor 3 | Factor 4 | Factor 5 | Factor 6 |
|  | Substance Exposure | Obstetric Complications | Birth Complications | Postnatal Challenges | Parental Age | Medical Needs |
| Alcohol (Early) | **0.76** | -0.04 | 0.04 | -0.03 | **0.36** | 0.02 |
| Tobacco | **0.74** | 0.10 | -0.01 | 0.08 | -0.03 | -0.16 |
| Cannabis | **0.77** | 0.03 | 0.00 | 0.01 | -0.05 | -0.21 |
| Alcohol (Late) | **0.79** | -0.15 | -0.01 | -0.03 | **0.49** | 0.09 |
| Unexpected Pregnancy | **0.37** | 0.09 | 0.02 | 0.01 | -0.21 | -0.27 |
| Nausea | -0.05 | **0.32** | 0.07 | -0.01 | -0.23 | 0.01 |
| Bleeding | -0.04 | **0.45** | 0.11 | 0.10 | -0.02 | 0.13 |
| (Pre)eclampsia | -0.01 | **0.91** | -0.10 | 0.11 | 0.02 | -0.13 |
| Gall Bladder | 0.03 | **0.54** | 0.03 | -0.18 | -0.13 | 0.07 |
| Anemia | 0.04 | **0.40** | 0.17 | -0.06 | -0.25 | 0.01 |
| Urinary Tract Infection | 0.06 | **0.31** | 0.11 | -0.04 | -0.28 | 0.02 |
| Diabetes | 0.01 | **0.44** | 0.02 | -0.02 | 0.17 | 0.07 |
| High Blood Pressure | -0.05 | **0.82** | -0.04 | 0.01 | 0.04 | -0.09 |
| Placental Problems | -0.08 | **0.31** | 0.16 | 0.07 | 0.04 | 0.15 |
| Accident/Injury | 0.08 | **0.38** | 0.15 | -0.11 | -0.03 | 0.10 |
| Blue at Birth | 0.01 | -0.05 | **0.94** | -0.04 | 0.01 | -0.02 |
| Slow Heart Rate | -0.02 | 0.08 | **0.74** | 0.03 | -0.05 | -0.04 |
| Difficulty Breathing | 0.00 | -0.02 | **0.89** | 0.04 | 0.00 | -0.03 |
| Required Oxygen | 0.00 | 0.02 | **0.63** | **0.50** | 0.03 | 0.05 |
| Premature | -0.03 | -0.02 | -0.01 | **0.78** | -0.04 | 0.03 |
| Birthweight | 0.06 | 0.16 | 0.02 | **0.76** | 0.01 | 0.02 |
| Incubation | -0.04 | -0.13 | 0.04 | **0.78** | -0.01 | -0.12 |
| Maternal Age | -0.03 | 0.14 | 0.03 | 0.01 | **0.94** | -0.01 |
| Paternal Age | -0.02 | 0.13 | 0.01 | 0.01 | **0.78** | -0.05 |
| Other Obstetric | 0.09 | 0.07 | 0.05 | 0.16 | 0.00 | **0.44** |
| Doctors Visits | -0.01 | 0.10 | -0.02 | 0.19 | -0.04 | **0.41** |
| Jaundice | 0.01 | 0.06 | 0.23 | 0.26 | -0.03 | 0.06 |
| Rh Incompatibility | 0.05 | -0.02 | 0.16 | 0.04 | 0.04 | 0.07 |
| Prenatal Vitamins | 0.07 | 0.02 | 0.04 | -0.01 | 0.00 | -0.53 |
| Breastfed | -0.21 | -0.12 | 0.07 | -0.16 | 0.13 | 0.08 |
| C-Section | 0.06 | 0.27 | -0.06 | 0.25 | 0.15 | 0.17 |
| Bold indicates factor loadings >= 0.30; Grey indicates factor loadings <= 0.30 | | | | | | |

| **Supplemental Table 7. Model Fit Statistics for the Series of 10 Exploratory Factor Analyses** | | | | | | | |
| --- | --- | --- | --- | --- | --- | --- | --- |
|  | X^2^ | *df* | *p* | RMSEA | CFI | TLI | SRMR |
| 1 Factor | 14834.96 | 434 | <.001 | 0.054 | 0.56 | 0.53 | 0.142 |
| 2 Factors | 7903.91 | 404 | <.001 | 0.04 | 0.77 | 0.73 | 0.118 |
| 3 Factors | 5909.24 | 375 | <.001 | 0.036 | 0.83 | 0.79 | 0.107 |
| 4 Factors | 3562.37 | 347 | <.001 | 0.028 | 0.9 | 0.87 | 0.086 |
| 5 Factors | 2342.95 | 320 | <.001 | 0.024 | 0.94 | 0.91 | 0.061 |
| 6 Factors | 1585.87 | 294 | <.001 | 0.02 | 0.96 | 0.94 | 0.057 |
| 7 Factors | 1081.5 | 269 | <.001 | 0.016 | 0.98 | 0.96 | 0.053 |
| 8 Factors | 682.89 | 245 | <.001 | 0.013 | 0.99 | 0.97 | 0.04 |
| 9 Factors | 487.24 | 222 | <.001 | 0.01 | 0.99 | 0.98 | 0.034 |
| 10 Factors | 402.45 | 200 | <.001 | 0.009 | 0.99 | 0.99 | 0.029 |

| **Supplemental Table 9. Bivariate Correlations Between Perinatal Insult Dimensions** | | | | | | |
| --- | --- | --- | --- | --- | --- | --- |
|  | F1 | F2 | F3 | F4 | F5 | F6 |
|  | Substance Exposure | Obstetric Complications | Birth Complications | Postnatal Challenges | Parental Age | Medical Needs |
| F1 | 1 |  |  |  |  |  |
| F2 | 0.10* | 1 |  |  |  |  |
| F3 | 0.09* | 0.18* | 1 |  |  |  |
| F4 | -0.06 | 0.33* | 0.18* | 1 |  |  |
| F5 | -0.35* | -0.12 | -0.09* | 0.02 | 1 |  |
| F6 | 0.02 | 0.10* | 0.11* | 0.13* | 0.22* | 1 |
| *=significant at p<0.05 | | | | | | |

| **Supplemental Table 9: Results from Latent Growth Models for Psychotic-Like Experiences (Sum Scores)** | | | | | | | | | |
| --- | --- | --- | --- | --- | --- | --- | --- | --- | --- |
|  | **Raw Estimates** | | | | | **Fully Standardized Estimates** | | | |
|  | X^2^(40)=297.82; RMSEA=0.02; CFI=0.96; TLI=0.94; SRMR=0.03 | | | | | | | | |
|  | B [95% CI] | | P | | | B [95% CI] | | P | |
| Baseline | 4.98 [3.90-6.06] | | <0.001 | | | 1.96 [1.52-2.39] | | <0.001 | |
| Slope | -0.62 [-0.97- -0.28] | | <0.001 | | | -1.06 [-1.65- -0.47] | | <0.001 | |
| Year-Four | 2.49 [1.55-3.43] | | <0.001 | | | 1.27 [0.78-1.76] | | <0.001 | |
| Intercept Variance | 2.48 [2.37-2.58] | | <0.001 | | | 0.97 [0.97-0.98] | | <0.001 | |
| Slope Variance | 0.57 [0.52-0.62] | | <0.001 | | | 0.97 [0.96-0.98] | | <0.001 | |
| Year-Four Variance | 1.88 [1.75-2.01] | | <0.001 | | | 0.96 [0.95-0.97] | | <0.001 | |
|  | **Psychotic-Like Experiences** | | | | |  | | | |
|  | **Baseline** | **Slope** | | **Year-Four** | | **Baseline** | **Slope** | | **Year-Four** |
| Substance Exposure | 0.17***  [0.10-0.24] | -0.01  [-0.04-0.01] | | 0.11**  [0.04-0.18] | | 0.07***  [0.04-0.10] | -0.03  [-0.07-0.02] | | 0.06**  [0.02-0.09] |
| Obstetric Complications | 0.12**  [0.34-0.21] | -0.02  [-0.05-0.01] | | 0.03  [-0.04-0.10] | | 0.05**  [0.01-0.08] | -0.04  [-0.09-0.01] | | 0.02  [-0.02-0.05] |
| Birth Complications | 0.01  [-0.06-0.09] | 0.01  [-0.02-0.03] | | 0.04  [-0.03-0.11] | | 0.01  [-0.02-0.03] | 0.01  [-0.03-0.05] | | 0.02  [-0.01-0.05] |
| Postnatal Challenges | 0.05  [-0.03-0.14] | 0.01  [-0.02-0.03] | | 0.07*  [0.01-0.14] | | 0.02  [-0.01-0.05] | 0.01  [-0.03-0.05] | | 0.04*  [0.003-0.072] |
| Parental Age | 0.36***  [0.28-0.43] | -0.02  [-0.05- 0.00] | | 0.27**  [0.20-0.33] | | 0.14***  [0.11-0.17] | -0.04  [-0.08-0.01] | | 0.14***  [0.11-0.17] |
| Medical Needs | 0.06  [-0.03-0.14] | -0.04**  [-0.06- -0.01] | | -0.10**  [-0.16- -0.04] | | 0.02  [-0.01-0.06] | -0.07**  [-0.11- -0.02] | | -0.05**  [-0.08- -0.02] |
| *** p<0.001; ** p<0.01, * p<0.05 | | | | |  | | | | |

| **Supplemental Table 10: Comparison of the Magnitude of Standardized Effects** | | | | | | | | | |
| --- | --- | --- | --- | --- | --- | --- | --- | --- | --- |
|  | **Baseline** |  |  | **Slope** |  |  | **Year-Four** |  |  |
| Substance Exposure | Externalizing > Internalizing > PLE | | | Internalizing > Externalizing > PLE | | | Externalizing > Internalizing > PLE | | |
| Obstetric Complications | Internalizing > Externalizing > PLE | | | Internalizing > Externalizing > PLE | | | Internalizing > Externalizing > PLE | | |
| Birth Complications | Internalizing = Externalizing > PLE | | | PLE > Internalizing > Externalizing | | | PLE > Internalizing > Externalizing | | |
| Postnatal Challenges | PLE > Externalizing > Internalizing | | | PLE > Internalizing = Externalizing | | | PLE > Externalizing > Internalizing | | |
| Parental Age | PLE = Externalizing > Internalizing | | | PLE > Internalizing = Externalizing | | | PLE > Externalizing > Internalizing | | |
| Medical Needs | PLE > Externalizing > Internalizing | | | Internalizing = Externalizing > PLE | | | Externalizing > Internalizing > PLE | | |
| Note: Comparisons are based on qualitative differences in mean beta estimates from fully standardized results (Supplementary Table 6). | | | | | | | | | |

| **Supplemental Table 11. Substance Exposure Dimension Tertiles and Psychotic-Like Experiences.** | | | | |
| --- | --- | --- | --- | --- |
| Variable | Tertile | Point Estimate | Lower Confidence Interval | Upper Confidence Interval |
| Baseline (T0) | **Bottom** | -0.033 | -0.050 | -0.016 |
|  | Middle | 0.007 | -0.011 | 0.024 |
|  | **Upper** | 0.024 | 0.006 | 0.042 |
| Slope | Bottom | 0.005 | -0.007 | 0.016 |
|  | Middle | -0.005 | -0.017 | 0.007 |
|  | Upper | 0.004 | -0.008 | 0.017 |
| Year-Four | Bottom | -0.019 | -0.050 | 0.011 |
|  | Middle | -0.009 | -0.041 | 0.022 |
|  | **Upper** | 0.037 | 0.004 | 0.070 |
| Bold indicates associations where the 95% Confidence Interval does not cross zero | | | | |

| **Supplemental Table 12. Obstetric Complications Dimension Tertiles and Psychotic-Like Experiences** | | | | |
| --- | --- | --- | --- | --- |
| Variable | Group | Point Estimate | Lower Confidence Interval | Upper Confidence Interval |
| Baseline (T0) | **Bottom** | -0.061 | -0.077 | -0.044 |
|  | Middle | -0.004 | -0.022 | 0.013 |
|  | **Upper** | 0.062 | 0.044 | 0.081 |
| Slope | Bottom | 0.007 | -0.004 | 0.018 |
|  | Middle | 0.005 | -0.006 | 0.017 |
|  | Upper | -0.010 | -0.023 | 0.003 |
| Year-Four | **Bottom** | -0.038 | -0.068 | -0.009 |
|  | Middle | 0.012 | -0.019 | 0.044 |
|  | Upper | 0.033 | -0.001 | 0.066 |
| Bold indicates associations where the 95% Confidence Interval does not cross zero | | | | |

| **Supplemental Table 13. Birth Complications Dimension Tertiles and Psychotic-Like Experiences** | | | | |
| --- | --- | --- | --- | --- |
| Variable | Group | Point Estimate | Lower Confidence Interval | Upper Confidence Interval |
| Baseline (T0) | **Bottom** | -0.123 | -0.138 | -0.109 |
|  | **Middle** | 0.035 | 0.017 | 0.053 |
|  | **Upper** | 0.088 | 0.069 | 0.108 |
| Slope | Bottom | 0.009 | -0.001 | 0.019 |
|  | Middle | -0.001 | -0.013 | 0.012 |
|  | Upper | -0.004 | -0.018 | 0.009 |
| Year-Four | **Bottom** | -0.096 | -0.122 | -0.070 |
|  | **Middle** | 0.033 | 0.001 | 0.066 |
|  | **Upper** | 0.075 | 0.040 | 0.111 |
| Bold indicates associations where the 95% Confidence Interval does not cross zero | | | | |

| **Supplemental Table 14. Postnatal Infant Challenges Dimension Tertiles and Psychotic-Like Experiences** | | | | |
| --- | --- | --- | --- | --- |
| Variable | Group | Point Estimate | Lower Confidence Interval | Upper Confidence Interval |
| Baseline (T0) | **Bottom** | -0.102 | -0.117 | -0.087 |
|  | **Middle** | -0.021 | -0.038 | -0.004 |
|  | **Upper** | 0.124 | 0.104 | 0.145 |
| Slope | Bottom | 0.005 | -0.005 | 0.015 |
|  | Middle | 0.008 | -0.003 | 0.020 |
|  | Upper | -0.009 | -0.023 | 0.005 |
| Year-Four | **Bottom** | -0.087 | -0.114 | -0.060 |
|  | Middle | 0.004 | -0.027 | 0.035 |
|  | **Upper** | 0.098 | 0.061 | 0.134 |
| Bold indicates associations where the 95% Confidence Interval does not cross zero | | | | |

| **Supplemental Table 15. Parental Age Dimension Tertiles and Psychotic-Like Experiences** | | | | |
| --- | --- | --- | --- | --- |
| Variable | Group | Point Estimate | Lower Confidence Interval | Upper Confidence Interval |
| Baseline (T0) | **Bottom** | -0.096 | -0.111 | -0.081 |
|  | **Middle** | -0.046 | -0.063 | -0.029 |
|  | **Upper** | 0.145 | 0.125 | 0.166 |
| Slope | Bottom | 0.003 | -0.007 | 0.013 |
|  | Middle | 0.0002 | -0.011 | 0.012 |
|  | Upper | 0.004 | -0.010 | 0.018 |
| Year-Four | **Bottom** | -0.088 | -0.115 | -0.061 |
|  | **Middle** | -0.045 | -0.076 | -0.015 |
|  | **Upper** | 0.158 | 0.121 | 0.195 |
| Bold indicates associations where the 95% Confidence Interval does not cross zero | | | | |

| **Supplemental Table 16. Medical Needs Dimension Tertiles and Psychotic-Like Experiences** | | | | |
| --- | --- | --- | --- | --- |
| Variable | Group | Point Estimate | Lower Confidence Interval | Upper Confidence Interval |
| Baseline (T0) | **Bottom** | -0.025 | -0.041 | -0.008 |
|  | **Middle** | -0.024 | -0.042 | -0.007 |
|  | **Upper** | 0.047 | 0.028 | 0.065 |
| Slope | Bottom | 0.006 | -0.006 | 0.017 |
|  | Middle | 0.014 | 0.002 | 0.026 |
|  | **Upper** | -0.016 | -0.029 | -0.004 |
| Year-Four | Bottom | -0.008 | -0.038 | 0.023 |
|  | Middle | 0.017 | -0.015 | 0.048 |
|  | Upper | -0.001 | -0.034 | 0.032 |
| Bold indicates associations where the 95% Confidence Interval does not cross zero | | | | |

| **Supplemental Table 17. Substance Exposure Dimension Tertiles and Internalizing Symptoms** | | | | |
| --- | --- | --- | --- | --- |
| Variable | Group | Point Estimate | Lower Confidence Interval | Upper Confidence Interval |
| Baseline (T0) | **Bottom** | -0.063 | -0.080 | -0.047 |
|  | **Middle** | -0.042 | -0.059 | -0.025 |
|  | **Upper** | 0.107 | 0.088 | 0.125 |
| Slope | Bottom | -0.006 | -0.018 | 0.005 |
|  | Middle | 0.005 | -0.007 | 0.017 |
|  | Upper | 0.001 | -0.011 | 0.014 |
| Year-Four | **Bottom** | -0.083 | -0.112 | -0.053 |
|  | Middle | -0.027 | -0.058 | 0.004 |
|  | **Upper** | 0.110 | 0.077 | 0.144 |
| Bold indicates associations where the 95% Confidence Interval does not cross zero | | | | |

| **Supplemental Table 18. Obstetric Complications Dimension Tertiles and Internalizing Symptoms** | | | | |
| --- | --- | --- | --- | --- |
| Variable | Group | Point Estimate | Lower Confidence Interval | Upper Confidence Interval |
| Baseline (T0) | **Bottom** | -0.099 | -0.115 | -0.083 |
|  | **Middle** | -0.027 | -0.044 | -0.011 |
|  | **Upper** | 0.126 | 0.107 | 0.145 |
| Slope | Bottom | 0.006 | -0.005 | 0.017 |
|  | Middle | 0.004 | -0.007 | 0.016 |
|  | Upper | -0.011 | -0.024 | 0.002 |
| Year-Four | **Bottom** | -0.082 | -0.111 | -0.053 |
|  | Middle | -0.015 | -0.046 | 0.016 |
|  | **Upper** | 0.093 | 0.059 | 0.127 |
| Bold indicates associations where the 95% Confidence Interval does not cross zero | | | | |

| **Supplemental Table 19. Birth Complications Dimension Tertiles and Internalizing Symptoms** | | | | |
| --- | --- | --- | --- | --- |
| Variable | Group | Point Estimate | Lower Confidence Interval | Upper Confidence Interval |
| Baseline (T0) | **Bottom** | -0.094 | -0.110 | -0.078 |
|  | **Middle** | -0.035 | -0.052 | -0.018 |
|  | **Upper** | 0.132 | 0.112 | 0.151 |
| Slope | **Bottom** | 0.018 | 0.008 | 0.029 |
|  | Middle | -0.001 | -0.013 | 0.010 |
|  | **Upper** | -0.018 | -0.031 | -0.005 |
| Year-Four | **Bottom** | -0.039 | -0.067 | -0.011 |
|  | **Middle** | -0.039 | -0.069 | -0.008 |
|  | **Upper** | 0.079 | 0.044 | 0.114 |
| Bold indicates associations where the 95% Confidence Interval does not cross zero | | | | |

| **Supplemental Table 20. Postnatal Challenges Dimension Tertiles and Internalizing Symptoms** | | | | |
| --- | --- | --- | --- | --- |
| Variable | Group | Point Estimate | Lower Confidence Interval | Upper Confidence Interval |
| Baseline (T0) | Bottom | -0.010 | -0.027 | 0.007 |
|  | **Middle** | -0.062 | -0.079 | -0.045 |
|  | **Upper** | 0.073 | 0.055 | 0.091 |
| Slope | Bottom | 0.008 | -0.003 | 0.020 |
|  | Middle | 0.006 | -0.005 | 0.018 |
|  | **Upper** | -0.016 | -0.028 | -0.003 |
| Year-Four | Bottom | 0.015 | -0.015 | 0.046 |
|  | **Middle** | -0.043 | -0.073 | -0.013 |
|  | Upper | 0.026 | -0.008 | 0.060 |
| Bold indicates associations where the 95% Confidence Interval does not cross zero | | | | |

| **Supplemental Table 21. Parental Age Dimension Tertiles and Internalizing Symptoms** | | | | |
| --- | --- | --- | --- | --- |
| Variable | Group | Point Estimate | Lower Confidence Interval | Upper Confidence Interval |
| Baseline (T0) | **Bottom** | -0.059 | -0.075 | -0.042 |
|  | Middle | -0.010 | -0.027 | 0.007 |
|  | **Upper** | 0.071 | 0.052 | 0.090 |
| Slope | **Bottom** | 0.023 | 0.012 | 0.034 |
|  | Middle | 0.002 | -0.010 | 0.013 |
|  | **Upper** | -0.026 | -0.039 | -0.013 |
| Year-Four | Bottom | 0.009 | -0.020 | 0.039 |
|  | Middle | -0.005 | -0.035 | 0.026 |
|  | Upper | -0.007 | -0.041 | 0.028 |
| Bold indicates associations where the 95% Confidence Interval does not cross zero | | | | |

| **Supplemental Table 22. Medical Needs Dimension Tertiles and Internalizing Symptoms** | | | | |
| --- | --- | --- | --- | --- |
| Variable | Group | Point Estimate | Lower Confidence Interval | Upper Confidence Interval |
| Baseline (T0) | **Bottom** | -0.063 | -0.079 | -0.047 |
|  | Middle | 0.010 | -0.008 | 0.028 |
|  | **Upper** | 0.053 | 0.035 | 0.071 |
| Slope | Bottom | 0.004 | -0.007 | 0.015 |
|  | Middle | -0.001 | -0.013 | 0.011 |
|  | Upper | -0.004 | -0.016 | 0.008 |
| Year-Four | **Bottom** | -0.052 | -0.081 | -0.022 |
|  | Middle | 0.007 | -0.025 | 0.039 |
|  | **Upper** | 0.041 | 0.008 | 0.073 |
| Bold indicates associations where the 95% Confidence Interval does not cross zero | | | | |

| **Supplemental Table 23. Substance Exposure Dimension Tertiles and Externalizing Symptoms** | | | | |
| --- | --- | --- | --- | --- |
| Variable | Group | Point Estimate | Lower Confidence Interval | Upper Confidence Interval |
| Baseline (T0) | **Bottom** | -0.093 | -0.109 | -0.077 |
|  | **Middle** | -0.049 | -0.066 | -0.032 |
|  | **Upper** | 0.142 | 0.123 | 0.161 |
| Slope | Bottom | 0.004 | -0.007 | 0.014 |
|  | Middle | 0.008 | -0.003 | 0.019 |
|  | Upper | -0.009 | -0.022 | 0.004 |
| Year-Four | **Bottom** | -0.083 | -0.111 | -0.054 |
|  | Middle | -0.025 | -0.055 | 0.005 |
|  | **Upper** | 0.115 | 0.080 | 0.150 |
| Bold indicates associations where the 95% Confidence Interval does not cross zero | | | | |

| **Supplemental Table 24. Obstetric Complications Dimension Tertiles and Externalizing Symptoms** | | | | |
| --- | --- | --- | --- | --- |
| Variable | Group | Point Estimate | Lower Confidence Interval | Upper Confidence Interval |
| Baseline (T0) | **Bottom** | -0.095 | -0.110 | -0.079 |
|  | Middle | -0.006 | -0.023 | 0.011 |
|  | **Upper** | 0.099 | 0.080 | 0.118 |
| Slope | Bottom | 0.007 | -0.004 | 0.018 |
|  | Middle | 0.001 | -0.010 | 0.013 |
|  | Upper | -0.007 | -0.020 | 0.006 |
| Year-Four | **Bottom** | -0.073 | -0.102 | -0.045 |
|  | Middle | -0.002 | -0.033 | 0.029 |
|  | **Upper** | 0.078 | 0.044 | 0.112 |
| Bold indicates associations where the 95% Confidence Interval does not cross zero | | | | |

| **Supplemental Table 25. Birth Complications Dimension Tertiles and Externalizing Symptoms** | | | | |
| --- | --- | --- | --- | --- |
| Variable | Group | Point Estimate | Lower Confidence Interval | Upper Confidence Interval |
| Baseline (T0) | **Bottom** | -0.143 | -0.158 | -0.128 |
|  | Middle | 0.012 | -0.006 | 0.030 |
|  | **Upper** | 0.132 | 0.113 | 0.152 |
| Slope | **Bottom** | 0.019 | 0.009 | 0.028 |
|  | Middle | 0.004 | -0.008 | 0.016 |
|  | **Upper** | -0.020 | -0.033 | -0.006 |
| Year-Four | **Bottom** | -0.088 | -0.114 | -0.061 |
|  | Middle | 0.024 | -0.008 | 0.056 |
|  | **Upper** | 0.074 | 0.038 | 0.109 |
| Bold indicates associations where the 95% Confidence Interval does not cross zero | | | | |

| **Supplemental Table 26. Postnatal Challenges Dimension Tertiles and Externalizing Symptoms** | | | | |
| --- | --- | --- | --- | --- |
| Variable | Group | Point Estimate | Lower Confidence Interval | Upper Confidence Interval |
| Baseline (T0) | **Bottom** | -0.071 | -0.087 | -0.054 |
|  | **Middle** | -0.056 | -0.072 | -0.040 |
|  | **Upper** | 0.128 | 0.108 | 0.147 |
| Slope | **Bottom** | 0.014 | 0.003 | 0.025 |
|  | Middle | 0.003 | -0.008 | 0.014 |
|  | **Upper** | -0.014 | -0.027 | -0.001 |
| Year-Four | **Bottom** | -0.030 | -0.059 | -0.001 |
|  | **Middle** | -0.047 | -0.077 | -0.018 |
|  | **Upper** | 0.085 | 0.050 | 0.121 |
| Bold indicates associations where the 95% Confidence Interval does not cross zero | | | | |

| **Supplemental Table 27. Parental Age Dimension Tertiles and Externalizing Symptoms** | | | | |
| --- | --- | --- | --- | --- |
| Variable | Group | Point Estimate | Lower Confidence Interval | Upper Confidence Interval |
| Baseline (T0) | **Bottom** | -0.116 | -0.131 | -0.101 |
|  | **Middle** | -0.050 | -0.066 | -0.033 |
|  | **Upper** | 0.170 | 0.150 | 0.190 |
| Slope | **Bottom** | 0.022 | 0.012 | 0.032 |
|  | Middle | 0.005 | -0.006 | 0.016 |
|  | **Upper** | -0.023 | -0.037 | -0.009 |
| Year-Four | **Bottom** | -0.051 | -0.078 | -0.024 |
|  | **Middle** | -0.035 | -0.065 | -0.006 |
|  | **Upper** | 0.102 | 0.065 | 0.140 |
| Bold indicates associations where the 95% Confidence Interval does not cross zero | | | | |

| **Supplemental Table 28. Medical Needs Dimension Tertiles and Externalizing Symptoms** | | | | |
| --- | --- | --- | --- | --- |
| Variable | Group | Point Estimate | Lower Confidence Interval | Upper Confidence Interval |
| Baseline (T0) | **Bottom** | -0.025 | -0.041 | -0.008 |
|  | Middle | -0.005 | -0.022 | 0.013 |
|  | **Upper** | 0.028 | 0.010 | 0.046 |
| Slope | Bottom | 0.004 | -0.008 | 0.015 |
|  | Middle | -0.001 | -0.012 | 0.011 |
|  | Upper | -0.002 | -0.014 | 0.011 |
| Year-Four | Bottom | -0.013 | -0.044 | 0.018 |
|  | Middle | -0.007 | -0.038 | 0.025 |
|  | Upper | 0.023 | -0.009 | 0.055 |
| Bold indicates associations where the 95% Confidence Interval does not cross zero | | | | |

| **Figure S1. Scree plot from exploratory factor analysis** |
| --- |
| 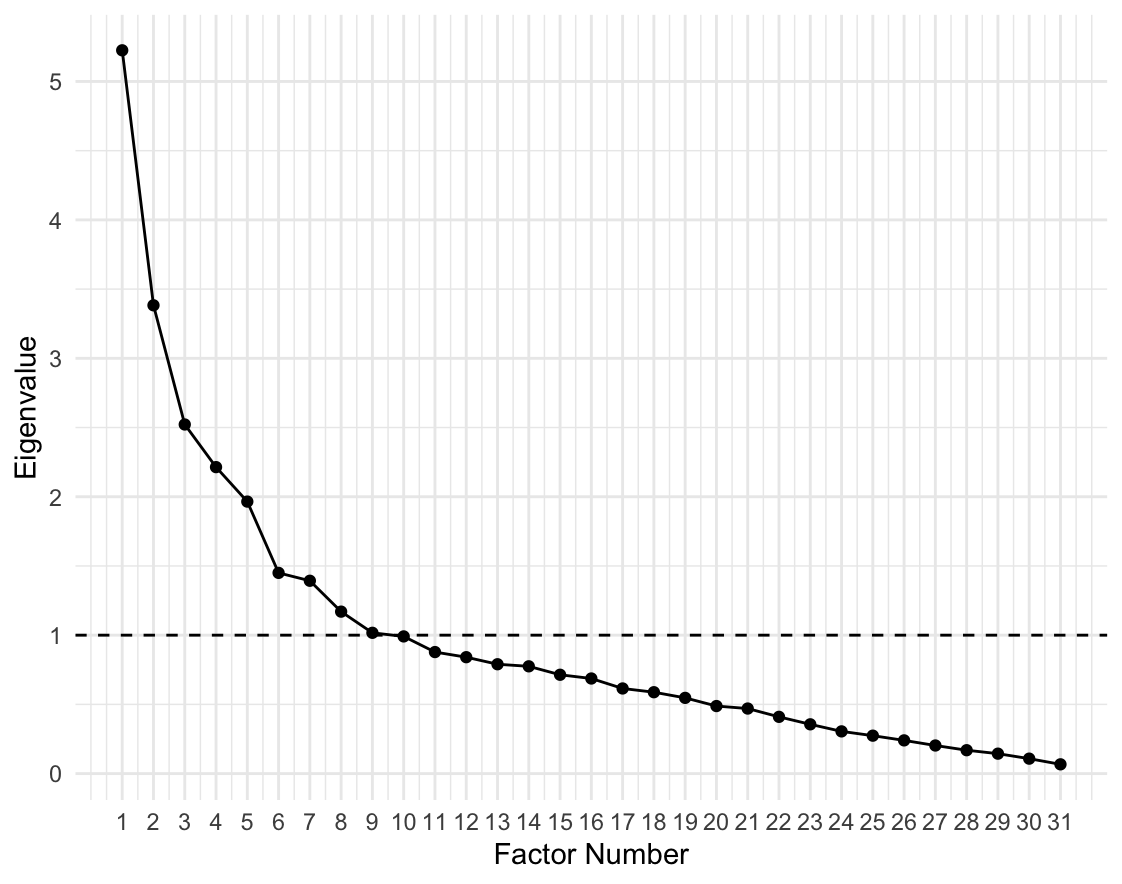 |

| **Supplemental Figure 2. Sample-level trajectories of (A) internalizing and (B) externalizing symptoms.** |
| --- |
| 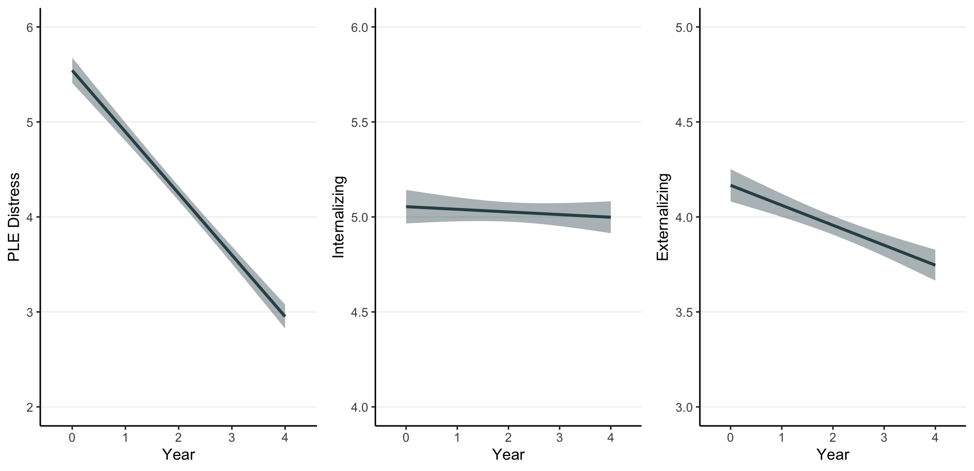 |
|  |
|  |

| **Supplemental Figure 3. Linear relationships between perinatal insult dimension tertiles and trajectories of internalizing symptoms.**   |
| --- |
|  |
|  |
| Note: Baseline, slopes, and intercepts correspond to Supplementary Tables 17-22. |

| **Supplemental Figure 4. Linear relationships between perinatal insult dimension tertiles and trajectories of externalizing symptoms.** |
| --- |
|  |
|  |
| Note: Baseline, slopes, and intercepts correspond to Supplementary Tables 23-28. |

**References**

1. Garavan, H. *et al.* Recruiting the ABCD sample: Design considerations and procedures. *Dev. Cogn. Neurosci.* **32**, 16–22 (2018).

2. Karcher, N. R. & Barch, D. M. The ABCD study: understanding the development of risk for mental and physical health outcomes. *Neuropsychopharmacology* **46**, 131–142 (2021).

3. Barch, D. M. *et al.* Demographic, physical and mental health assessments in the adolescent brain and cognitive development study: Rationale and description. *Dev. Cogn. Neurosci.* **32**, 55–66 (2018).

4. Iacono, W. G. *et al.* The utility of twins in developmental cognitive neuroscience research: How twins strengthen the ABCD research design. *Dev. Cogn. Neurosci.* **32**, 30–42 (2018).

5. Casey, B. J. *et al.* The Adolescent Brain Cognitive Development (ABCD) study: Imaging acquisition across 21 sites. *Dev. Cogn. Neurosci.* **32**, 43–54 (2018).

6. Rice, J. P. *et al.* Comparison of Direct Interview and Family History Diagnoses of Alcohol Dependence. *Alcohol. Clin. Exp. Res.* **19**, 1018–1023 (1995).

7. McLaughlin, K. A., Weissman, D. G. & Flournoy, J. Challenges with Latent Variable Approaches to Operationalizing Dimensions of Childhood adversity – a Commentary on Sisitsky et al. (2023). *Res. Child Adolesc. Psychopathol.* (2023) doi:10.1007/s10802-023-01114-4.

8. Hanafiah, M. H. Formative Vs. Reflective Measurement Model: Guidelines for Structural Equation Modeling Research. *Int. J. Anal. Appl.* (2020) doi:10.28924/2291-8639-18-2020-876.

9. Russell, J. D., Heyn, S. A., Peverill, M., DiMaio, S. & Herringa, R. J. Traumatic and Adverse Childhood Experiences and Developmental Differences in Psychiatric Risk. *JAMA Psychiatry* (2024) doi:10.1001/jamapsychiatry.2024.3231.

10. Nikolaidis, A. *et al.* Heterogeneity in caregiving-related early adversity: Creating stable dimensions and subtypes. *Dev. Psychopathol.* **34**, 621–634 (2022).

11. Brieant, A. *et al.* Characterizing the dimensional structure of early-life adversity in the Adolescent Brain Cognitive Development (ABCD) Study. *Dev. Cogn. Neurosci.* **61**, 101256 (2023).

12. Nweze, T., Ezenwa, M., Ajaelu, C. & Okoye, C. Childhood mental health difficulties mediate the long‐term association between early‐life adversity at age 3 and poorer cognitive functioning at ages 11 and 14. *J. Child Psychol. Psychiatry* **64**, 952–965 (2023).

13. Greenacre, M. *et al.* Principal component analysis. *Nat. Rev. Methods Primer* **2**, 100 (2022).

14. Orendain, N., Anderson, A., Galván, A., Bookheimer, S. & Chung, P. J. A data-driven approach to categorizing early life adversity exposure in the ABCD Study. *BMC Med. Res. Methodol.* **23**, 164 (2023).

15. Cudeck, R. Exploratory Factor Analysis. in *Handbook of Applied Multivariate Statistics and Mathematical Modeling* 265–296 (Elsevier, 2000). doi:10.1016/B978-012691360-6/50011-2.

16. Jeong, H. J. *et al.* Early life stress and functional network topology in children. *Dev. Cogn. Neurosci.* **66**, 101367 (2024).

17. Orri, M. *et al.* Perinatal adversity profiles and suicide attempt in adolescence and young adulthood: longitudinal analyses from two 20-year birth cohort studies. *Psychol. Med.* **52**, 1255–1267 (2022).

18. Lebel, C. A. *et al.* Characterizing adverse prenatal and postnatal experiences in children. *Birth Defects Res.* **111**, 848–858 (2019).

19. Marsh, H. W., Morin, A. J. S., Parker, P. D. & Kaur, G. Exploratory Structural Equation Modeling: An Integration of the Best Features of Exploratory and Confirmatory Factor Analysis. *Annu. Rev. Clin. Psychol.* **10**, 85–110 (2014).
